# Etiology-guided mutational signature learning from DNA repair knockouts in cell lines using supervised NMF

**DOI:** 10.1093/nargab/lqag078

**Published:** 2026-08-06

**Authors:** Sander Goossens, Yasin I Tepeli, Colm Seale, Joana P Gonçalves

**Affiliations:** Pattern Recognition & Bioinformatics, Intelligent Systems Department, EEMCS Faculty, Delft University of Technology, Van Mourik Broekmanweg 6, 2628 XE Delft, Netherlands; Pattern Recognition & Bioinformatics, Intelligent Systems Department, EEMCS Faculty, Delft University of Technology, Van Mourik Broekmanweg 6, 2628 XE Delft, Netherlands; Pattern Recognition & Bioinformatics, Intelligent Systems Department, EEMCS Faculty, Delft University of Technology, Van Mourik Broekmanweg 6, 2628 XE Delft, Netherlands; Holland Proton Therapy Center (HollandPTC), 2600 AC Delft, Netherlands; Pattern Recognition & Bioinformatics, Intelligent Systems Department, EEMCS Faculty, Delft University of Technology, Van Mourik Broekmanweg 6, 2628 XE Delft, Netherlands

## Abstract

Many tumours show deficiencies in DNA damage response (DDR), not only driving tumorigenesis but also exposing vulnerabilities with therapeutic potential. Assessing which patients might benefit from DDR-targeting therapy requires knowledge of tumour DDR deficiency (DDRd) status, with mutational signatures reportedly better predictors than loss-of-function mutations. Existing DDRd models offer effective prediction for pathways with well-characterized processes and mutational signatures. Nevertheless, development of models for additional DDRd and clinically relevant mechanisms could be hampered by the fact that most mutational signatures have unknown etiology. Using supervised non-negative matrix factorization (SNMF), we integrate mutational signature learning with multiclass DDR-deficiency prediction to enable etiology-guided learning of signatures from cell lines with confirmed gene knockouts. Applied to DDR gene knockout human-induced pluripotent stem cell lines, SNMF identified etiology-aligned representations of deficiency in homologous recombination, mismatch repair, and base excision repair. Even guided by pathway-level labels, SNMF captured gene-specific base excision repair submechanisms, showing the integration offered added granularity. Learned cell line signatures showed high similarity to tumour-derived COSMIC signatures, revealed associations with mutations in DDR genes, and enabled high recall of tumours with DDR deficiencies. We envision that SNMF-like methods could leverage knockout screens to learn etiology-guided signatures for improved DDRd annotation and treatment optimization. SNMF is available at: https://github.com/joanagoncalveslab/SNMF.

## Introduction

The accumulation of mutations caused by various DNA damage-inducing processes and associated DNA damage response (DDR) can lead to genome instability, an enabling hallmark of cancer [1] which can also be responsible for DDR pathway deficiencies (DDRd) frequently occurring in tumour cells. Tumour DDR or repair deficiencies provide opportunities for targeted treatment, namely by leveraging known synthetic lethalities where joint disruption of multiple genes leads to cell death but individual inactivation does not affect cell viability [2]. For example, patients with *BRCA1/2*-deficient tumours can benefit from therapy inhibiting the synthetic lethal partner gene *PARP1*.

To apply treatments targeting DDRd, it is necessary to identify which deficiencies might be present in the tumour. This could be done by detecting loss-of-function mutations in DDR-related genes. However, this approach has limited sensitivity since not all genes involved in every repair pathway are known, and gene function can be modulated via alternative mechanisms such as epigenetic regulation [3]. To capture downstream effects of alterations to DDR function, two models have been developed that predict repair deficiency based on patterns of mutations imprinted on the genomes of tumour cells: HRDetect [4] and MMRDetect [5]. Both models offer tailored and effective prediction of deficiency in a specific DDR pathway, respectively homologous recombination and mismatch repair, confirming that mutational signatures associated with processes of interest can be meaningfully integrated for DDRd prediction.

More generally, this pioneering work creates opportunities to develop new models for additional DDRds or even other mutational processes of clinical relevance. We identify some general challenges to achieve this. When exploiting existing mutational signatures for DDR prediction, the signatures are learned independently from the prediction model, thus are not directly optimized to distinguish deficiency or to disentangle contributions of different pathways and genes. As an example, the COSMIC catalogue of tumour-derived signatures contains seven partially redundant signatures associated with mismatch repair, without a clear etiological distinction. While expert curation can help select the most suitable signatures, it may not always be possible to discern relevance and role based on the available information. In effect, most tumour-derived mutational signatures remain unannotated to date, which limits their potential use for building new models. Training prediction models directly on tumour samples also poses additional challenges due to the lack of ground truth DDRd status. While proxies such as loss-of-function (LOF) mutations to known DDR genes can be used, evidence suggests that mutational signatures may be more comprehensive than canonical mutations at capturing the full range of mechanisms involved in a DDRd [4]. For this reason, learning DDRd predictors based on tumour samples with gene LOF labels could lead to suboptimal models.

We reason that supervised multiclass signature learning can leverage trusted knowledge of DDRd status to identify signatures that both (i) effectively predict a multiplicity of DDR deficiencies and (ii) capture more complete and representative patterns of each deficiency. Crucially, integrating DDRd status into the learning enables automated identification of meaningful signatures without manual annotation or preselection. The multiclass model further allows for learning and prediction of multiple DDR deficiencies, including cases where these might co-occur. When trained on samples with reliable DDRd labels, such as cell lines with induced DDR gene knockouts, supervised signature learning could systematically uncover DDR gene- and pathway-specific mutational patterns driving comprehensive and interpretable prediction. However, while cell lines provide controlled model systems for learning, they may not fully recapitulate the complexity of tumour mutagenesis. In contrast, tumour-derived signature catalogues are hard to supervise due to absence of reliable ground truth, need post-learning annotation, and might contain redundant or overlapping signatures. Deeper investigation into the conditions under which supervised signature learning from cell line data can meaningfully transfer to tumours is thus necessary. Ultimately, we aim to assess if DDRd-related signatures and prediction models can (i) be learned from cell line data with confirmed etiology and (ii) transferred to enable discovery or confirmation of etiological annotations for mutational processes underlying patient tumours.

Most mutational signatures have been discovered over the years using unsupervised learning, specifically non-negative matrix factorization (NMF) models [6]. Supervised NMF (SNMF) methods have been proposed in other fields, coupling NMF-based latent pattern extraction to various classification objectives [7], including Fisher discriminant regularization [8], Frobenius loss [9], or cross-entropy loss [10, 11]. One other method, supervised negative binomial NMF (SNBNMF), has been proposed to learn mutational signatures predictive of cancer subtype by integrating NMF with a support vector machine, relying on absolute mutation counts modelled using a negative binomial (NB) distribution [12].

**Figure 1.**
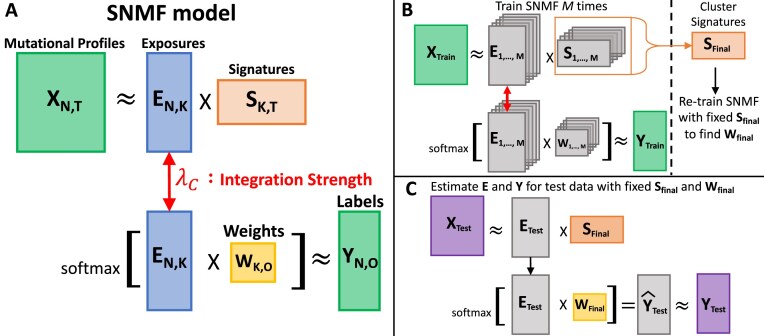
Overview of SNMF. (**A**) The SNMF model integrates (top) decomposition of mutation profiles into exposures and mutational signatures using NMF ($\boldsymbol{X} \approx \boldsymbol{E} \boldsymbol{S}$) and (bottom) exposure-based prediction of DNA repair deficiency using logistic regression (softmax$(\boldsymbol{E} \boldsymbol{W})$  $\approx \boldsymbol{Y}$). (**B** and **C**) SNMF training and testing: (B) train $M$ times to learn robust signatures $\boldsymbol{S_{final}}$ and retrain with fixed $\boldsymbol{S_{final}}$ to get regression weights $\boldsymbol{W_{final}}$; (C) obtain SNMF exposures and predictions for test data with fixed $\boldsymbol{S_{final}}$ and $\boldsymbol{W_{final}}$.

We explore a similar approach, using SNMF to integrate mutational signature learning and multiclass DDRd prediction into a single model (Fig. 1A). This is achieved through a weighted loss combining Frobenius reconstruction from NMF and cross-entropy from multinomial logistic regression. In contrast to SNBNMF, SNMF uses relative proportions or normalized counts, which do not follow a NB distribution. This choice is intentionally made because absolute counts are typically confounded with total mutation burden, which can vary widely across samples and thus pose challenges to cross-domain generalization. The use of relative proportions can be especially important for successful transfer between domains such as cell lines and tumours, genomes and exomes, or cancer types, where samples exhibit mutation burdens of vastly different magnitude. Additionally, we integrate SNMF into the SigProfiler framework [6] to enable hyperparameter optimization, and more robust and stable learning. We further derive update formulas for gradient-based optimization of SNMF and compare SNMF against existing models. As a proof-of-concept, we apply SNMF to mutation profiles of human-induced pluripotent stem (hiPS) cell lines with DDR gene knockouts and investigate its ability to identify mutational signatures predictive of DDRd. Finally, we assess transfer of the SNMF-learned cell line signatures and prediction model to patient tumour samples through similarity to tumour-derived signatures, association with mutation status of known DDR genes, and DDRd prediction in tumours.

## Materials and methods

### The SNMF model

#### Problem formulation

*Definitions*. We define a matrix of mutation profiles $ \boldsymbol{X} \in [0,1]^{N\times T}$, with $N$ and $T$ as numbers of samples and mutation types, respectively (Supplementary Section SA.1). An entry $x_{n,t}$ represents the relative frequency of mutation type $t$ in sample $n$, and each mutation profile (row in $\boldsymbol{X}$) is a probability distribution over all mutation types: $ \sum _{t=1}^T x_{n,t} = 1 , \forall n \in \lbrace 1, ..., N\rbrace $. We also define the respective matrix of DNA repair deficiency status $\boldsymbol{Y} \in \lbrace 0,1\rbrace ^{N\times O}$, over the $O$ different repair deficiencies or classes the model can predict. The $n^{th}$ row in $\boldsymbol{Y}$ represents the one-hot encoded repair deficiency status of sample $n$, where $\sum _{o=1}^O y_{n,o} = 1, \forall n \in \lbrace 1, ..., N\rbrace $.

*Problem*. Given a matrix of mutation profiles $\boldsymbol{X}$, the respective matrix of DNA repair deficiencies $\boldsymbol{Y}$, and a number of signatures $K \le \min (T,N)$, SNMF learns a model by iteratively optimizing (Fig. 1A): (i) mutation profiles in $\boldsymbol{X}$ as linear combinations of $K$ underlying mutational signatures (matrix $\boldsymbol{S}$) weighed by exposures (matrix $\boldsymbol{E}$) and (ii) a linear mapping between sample exposures $\boldsymbol{E}$ and the output class or DNA repair deficiency $\boldsymbol{Y}$ with learned weights $\boldsymbol{W}$. Once built, the SNMF model can be used to estimate signature exposures and predict repair deficiency for mutation profiles of unseen samples (Figs. 1B-1C).

#### Loss function

*Reconstruction loss*. To identify signatures, matrix $\boldsymbol{X}$ is decomposed as a product of matrices $\boldsymbol{S}$ and $\boldsymbol{E}$, $\boldsymbol{X} \approx \boldsymbol{E} \boldsymbol{S}$. The signatures matrix $\boldsymbol{S} \in [0,1]^{K\times T}$ contains the frequencies of each of the $T$ mutation types for each of the $K$ signatures identified across samples, whereas the exposure matrix $\boldsymbol{E} \in [0,1]^{N\times K}$ contains the contribution of each signature to each of the $N$ samples. We use NMF to find $\boldsymbol{S}$ and $\boldsymbol{E}$ [13], with multiplicative updates to optimize the Frobenius reconstruction error ($\mathcal {L}_r$, Supplementary Sections SA.2 and A.3).

*Classification loss*. For prediction of DDRd, SNMF learns a linear mapping between signature exposures $\boldsymbol{E}$ and DDRd status (class labels) $\boldsymbol{Y}$ using multinomial logistic regression. This estimates a matrix of weights $\boldsymbol{W} \in \mathbf {R}^{K\times O}$, with entry $w_{k,o}$ denoting the weight of signature $ k$ for DDRd output class $o$, by minimizing the categorical cross-entropy loss $\mathcal {L}_c$ between observed ($y$) and predicted ($\hat{y}$) status. To mitigate overfitting, we include L2 or ridge regularization, with strength controlled by hyperparameter $ \lambda _{L2}$ (Supplementary Sections SA.2 and A.4).

*Integrated (total) loss*. To integrate both aims, SNMF optimizes a combined loss (eq. 1), defined as a sum of the reconstruction ($ \mathcal {L}_{r}$) and classification ($ \mathcal {L}_{c}$) losses. The hyperparameter $ \lambda _c$ controls the integration between the two components: the larger the value, the larger the influence of the classification loss on exposures and signatures, while $ \lambda _c = 0$ results in non-integrated signature decomposition (NMF) and repair deficiency prediction.


(1) \begin{eqnarray*}
\mathcal {L}_{tot} = \mathcal {L}_r + \lambda _c \mathcal {L}_c
\end{eqnarray*}

*Multiplicative updates*. The total loss $\mathcal {L}_{tot}$ is minimized by iteratively applying gradient descent (GD) on $\mathcal {L}_{tot}$ with respect to $\boldsymbol{S, E}$, and $\boldsymbol{W}$ (Supplementary Section SA.2). The final updates are obtained by substituting the derivatives of the loss and the learning rates in the GD update formulas ($\odot$ and division are element-wise) (Supplementary Sections SA.2–A.8). The learning rate for the classifier weights ($\mu _W$) was set to 0.005.


(2) \begin{eqnarray*}
\boldsymbol{S} &\leftarrow \boldsymbol{S}\odot \frac{\boldsymbol{E}^T\boldsymbol{X}}{\boldsymbol{E}^T\boldsymbol{ES}}
\end{eqnarray*}

(3) \begin{eqnarray*}
\boldsymbol{E} & \leftarrow \boldsymbol{E}\odot \frac{\boldsymbol{XS}^T - \frac{\lambda _c}{2} (\boldsymbol{\hat{Y}} - \boldsymbol{Y}) \boldsymbol{W}^T}{\boldsymbol{ES}\boldsymbol{S}^T}
\end{eqnarray*}

(4) \begin{eqnarray*}
\boldsymbol{W} &\leftarrow \boldsymbol{W} - \mu _W (\boldsymbol{E}^T( \boldsymbol{\hat{Y}} - \boldsymbol{Y}) + 2\lambda _{L2} \boldsymbol{W})
\end{eqnarray*}

*Non-negativity*. The SNMF exposure update does not ensure non-negativity as a result of the subtraction between predicted and true labels ($\boldsymbol{\hat{Y}} - \boldsymbol{Y})$ (eq. 3). To ensure non-negativity, at each exposure update, negative values are set to a small positive value $ 10^{-25}$. We avoid using exactly 0 to prevent division by zero in the signature update (eq. 2). Each updated signature is normalized to keep it as a probability distribution over the $T$ mutation types.

### SNMF model learning and prediction

We implement SNMF as an extension of SigProfiler [6], which already offers regular NMF and a method to determine the optimal number of signatures. The application of SNMF comprises training (Fig. 1B) and testing (Fig. 1C), with input consisting of mutation profiles and DDR labels of train and test samples ($\boldsymbol{X, Y, X_{test}}$), and hyperparameters ($K, \lambda _c$, and $ \lambda _{L2}$).

The SNMF model is trained by randomly initializing $\boldsymbol{S}$ and $\boldsymbol{E}$, and then iteratively updating $\boldsymbol{S}$, $\boldsymbol{E}$ and $\boldsymbol{W}$ (eq. 2–4), resulting in a set of $K$ signatures with respective exposures and regression weights. To ensure robustness against variations caused by random initialization while maintaining computational efficiency, we repeat training $M=10$ times. The resulting $M \times K$ signatures found across runs are clustered using a variation of *K*-means (Fig. 1B). Specifically, we enforce each cluster is assigned one signature from each run, similar to the approach used in SigProfiler [6]. The final set of $K$ signatures $\boldsymbol{S_{final}}$ is obtained by taking the mean signatures within each cluster. In the second training step, $\boldsymbol{S_{final}}$ is fixed and fitted to obtain the exposures $\boldsymbol{E}$ and weights $\boldsymbol{W_{final}}$ (Fig. 1B) using non-integrated SNMF ($\lambda _c = 0$), meaning $\boldsymbol{E}$ is optimized with respect to $\mathcal {L}_r$, rather than labels $Y$, mimicking the test setting where labels are unavailable.

In test phase, $\boldsymbol{S_{final}}$ and $\boldsymbol{W_{final}}$ are fixed. For each unseen test sample in $\boldsymbol{X_{test}}$, corresponding exposures $\boldsymbol{E_{test}}$ are estimated using non-negative least squares (NNLS) based on reconstruction loss $\mathcal {L}_r$ (Fig. 1C). The test exposures $\boldsymbol{E_{test}}$, together with the classification weights $\boldsymbol{W_{final}}$ of the trained SNMF model, are used to calculate the prediction probability of each deficiency class for every test sample ($ \boldsymbol{\hat{Y}}$). The final predicted label is the class with the highest probability.

### Data and preprocessing

#### Cell line mutation profiles

Since tumours are hard to reliably label for DDRd and contain mutations from etiologies beyond the potential DDRd, we trained SNMF using data from [5], profiling mutations for hiPS cell lines with biallelic knockouts (KOs) of 42 different genes involved in 12 DNA repair pathways. Knockout of gene *ATP2B4* was also included as a control. There were 2 to 8 replicates per gene KO, resulting in 173 samples. Mutations were obtained after culturing for 15 days, whole-genome sequencing, alignment to the human reference GRCh37/hg19, and somatic mutation calling. We focused on 96 types of single base substitutions (SBS) comprising: the substitution itself, with six different types considering pyrimidines C and T as reference (G and A are complementary); and the two neighbouring 5′ and 3′ bases, resulting in 16 possible for each SBS. The original count-based mutation profiles were normalized to represent a probability distribution over the 96 SBS types. In line with [5], we selected gene KOs for which all replicates had either high mutation count and/or low cosine similarity with the average profile of control samples (Supplementary Figs S1 and S2). Because ATP2B4 knockout samples showed a C$> $A-rich mutation profile, we additionally performed a sensitivity analysis using mutation profiles corrected by removing the ATP2B4 profile, as in [5]. Given the challenge of disentangling which C$> $A counts arise from a genuine role of genes in the repair of oxidative damage, and which C$> $A counts arise due to cell culture effects (Supplementary Fig. S3), we report the main SNMF analysis using the original normalized profiles but then interpret the SBS mutation types overlapping with the ATP2B4 profile cautiously, particularly for BER-related signatures.

#### Cancer patient mutation profiles and signatures

Cell line signatures found by SNMF were compared to 78 cancer-related COSMIC signatures (v3.2). To assess SNMF prediction for patient tumours, we used whole-exome MC3 somatic mutations from The Cancer Genome Atlas (TCGA, [14]). For homologous recombination deficiency (HRd), we focused on breast cancer (BRCA), since HRd is most prevalent in BRCA and ovarian (OV) cancers [15], with a much larger number of BRCA tumours (791 versus 65 OV). For mismatch repair deficiency (MMRd), we evaluated SNMF prediction on the three tumour types with highest prevalence of microsatellite instability (MSI): uterine (UCEC), colon (COAD), and stomach (STAD) [16]. Mutational spectra samples from these tumour types also showed apparent clustering based on MLH1 mutation status, known to be strongly associated with MSI (Supplementary Fig. S4). We used repair status of patient tumours based on mutation status of 81 repair genes assigned to 9 pathways (Supplementary Table S1) [17]. Each was annotated as wild-type, or heterozygous or homozygous mutated based on variant allele frequency of LOF mutations or epigenetic silencing.

### Data augmentation and hyperparameter optimization

To build the SNMF model, we split the cell line data into disjoint train and test sets. The test set contained one replicate of each gene KO and two replicates of the control samples. The remaining train samples were split into three folds for cross-validated hyperparameter optimization, with replicates per gene KO evenly spread across folds (Supplementary Fig. S5). In addition to the original train-test split, we created 10 different additional train-test splits to assess the variability of final model performance following the same leakage-avoiding procedure, resulting in a total of 11 evaluation data splits.

*Data augmentation*. To improve SNMF model robustness, given the limited number of cell line samples, we augmented the data using bootstrapped oversampling. We avoided data leakage by performing the bootstrapping independently within each train fold, and within the test set, such that each bootstrapped sample was kept in the same train fold (or test set) of its original real sample. Each new bootstrapped sample was created by considering the mutation profile of a real sample as a probability distribution, based on which we drew the same number of mutations as in the real sample, with replacement. The counts of each bootstrapped sample were divided by the sum so that the entire mutation profile summed to 1. Per train fold, we bootstrapped 100 samples per class, which was found to be representative. For the test set, we bootstrapped 1000 samples per class to obtain more granular estimates, since prediction is more efficient than model learning.

*Hyperparameter optimization*. The SNMF model has the following parameters: number of signatures $K$, integration strength $\lambda _{c}$, and regularization strength $\lambda _{L2}$. Since the number of signatures $K$ had the most impact on performance, we first determined $K$ as the value that resulted in the highest accuracy without a decrease in stability across a range of $\lambda _{c}$ and $\lambda _{L2}$. Then, we selected $\lambda _{c}$ and $\lambda _{L2}$ together based on the optimal Pareto settings, that is, the hyperparameter value combinations for which no other combination yielded higher accuracy and stability. Using the optimal hyperparameter setting, we trained the final integrated SNMF model on the entire cell line train set.

### Evaluation of the SNMF model

The cell line-trained SNMF model was first evaluated and compared to benchmark models across the 11 cell line bootstrapped train-test data splits.

*Evaluation metrics*. We used three metrics: stability of the identified signatures, and both prediction accuracy and reconstruction error on the test samples. Signature stability or reproducibility was measured using the average of silhouette widths (ASW) of the $K$ signature clusters ($Cluster_1, ..., Cluster_K$) obtained with $M$ training runs, based on cosine distance between signatures. The (average) SW ranges between −1 and 1, with 1 indicating consistent signature(s) across runs and lower SW values denoting weaker reproducibility. We used accuracy to assess classification performance, corresponding to the fraction of correctly predicted test samples (true positives and true negatives), out of all test samples. The reconstruction error evaluated how well the mutation profiles of the test samples could be recovered from the exposures and (fixed) signatures, defined based on the Frobenius norm as follows: $||\boldsymbol{X_{test} - E_{test}S_{final}}||_{F}^2$. The lower the error, the more accurate the approximation to the original test data $\boldsymbol{X_{test}}$. Since choosing a larger number of signatures $K$ typically results in lower reconstruction error, owing to the higher model complexity, we considered the reconstruction subordinate to stability for measuring the quality of signature decomposition.

*Benchmark models*. We assessed the integrated SNMF model that jointly learns signatures and a prediction model ($\lambda _c > 0)$ against 3 benchmarks: (i) non-integrated signature learning and exposure-based prediction using SNMF with $\lambda _c = 0$, equivalent to existing approaches that first apply unsupervised NMF signature learning and then build an exposure-based classification model; (ii) an L2-regularized (optimal strength: 100) logistic regression (LR) model learned directly from the mutation profiles $\boldsymbol{X}$, thus directly using the previously defined mutation profiles rather than signature exposures as input; and (iii) an L2-regularized (optimal strength: 0.01) LR model trained on exposures to COSMIC mutational signatures estimated for the cell line samples. For both LR models, we used scikit-learn LogisticRegression with L2 regularization, the saga solver, and hyperparameter optimization via GridSearchCV. All models were trained using the same train-test splits and bootstrapping procedures.

### Exposures and predictions for TCGA patient tumours

The final integrated SNMF model was used to obtain signature exposures and make DDRd predictions for TCGA patient tumour samples. We used recall to assess the ability to identify DDRd in tumours with a homozygous mutated core gene for the repair pathway of interest. We preferred recall to accuracy, since the absence of LOF mutations in a repair gene does not imply repair proficiency.

Given that genes involved in a repair pathway have different functions and could lead to distinct repair deficiency signatures, we also analysed the SNMF results for patient tumours on a gene rather than pathway level. To quantify the association between the mutation status of a repair gene and exposure to the corresponding SNMF deficiency signature, we performed a one sided Mann–Whitney *U* (MWU) test between the exposures ($E_{MMRd}$ or $E_{HRd}$) of mutated and non-mutated tumours, resulting respectively in statistics U1 and U2 together with the FDR-adjusted MWU *P*-value. We did not distinguish heterozygous and homozygous mutations, as very few tumours carried homozygous mutations for most genes. Because tumour mutation burden can confound associations between gene mutation status and signature exposure, we additionally performed an analysis of covariance (ANCOVA) for each gene, modelling signature exposure as a function of gene mutation status while including total SBS mutation count as a covariate. The resulting *P*-values for the mutation status term were FDR-adjusted across tested genes. We also quantified the co-occurrence of mutations for each pair of genes using Jaccard similarity.

For comparison, we also did the TCGA exposure and gene association analysis for the cell line signatures learned using the non-integrated setting ($\lambda _c = 0$). These signatures were fitted to the same tumour samples using the same procedure as for the integrated SNMF signatures, after which associations between tumour exposures and DDR–gene mutation status were tested using the same Mann–Whitney U framework.

## Results and discussion

### Gene knockouts with impact on the mutation profiles

**Figure 2.**
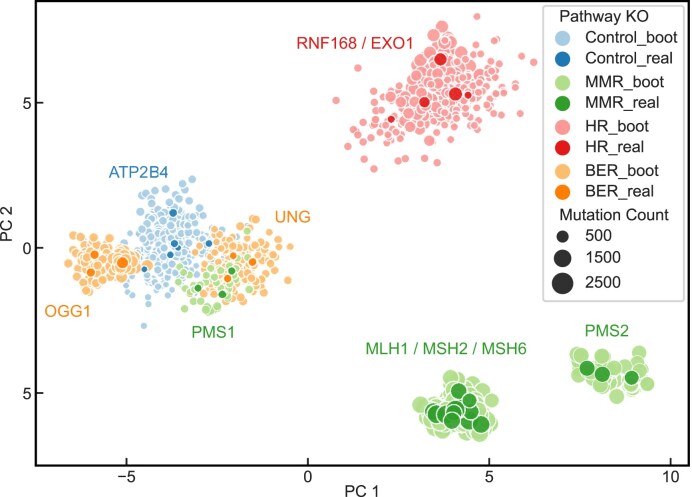
Gene knockout and bootstrapped training samples. Latent PCA plot of bootstrapped (light) and real (dark) cell line samples used for training.

Nine gene KOs showed mutation profiles distinguishable from the control samples (Fig. 2, Supplementary Fig. S2) and were annotated with a total of four repair pathways: mismatch repair (MMR: *MSH6, MSH2, MLH1, PMS2* and *PMS1*), base excision repair (BER: *UNG* and *OGG1*), homologous recombination (HR: *EXO1*), and double strand break repair (DSB: *RNF168*). The high similarity of *RNF168* and *EXO1* (cosine similarity 0.96) and literature supporting the role of *RNF168* in recruiting the *BRCA1–PALB2* complex to DNA damage for HR-directed repair [18] led us to annotate *RNF168* with HR as well. This resulted in four classes: MMRd, BERd, HRd, and control (*ATP2B4*). As expected, the bootstrapped samples clustered around the gene KO from which they were sampled (Fig. 2). We also confirmed that *RNF168* and *EXO1* indeed clustered together. On the other hand, *OGG1* and *UNG* formed their own clusters, indicative of distinct behaviour within BER. In addition, we observed that *PMS1* was not as discernable from the control samples as the other MMR gene KOs. The *PMS2* samples did not cluster together with the other MMR gene KOs, yet they were clearly distinct from the control samples.

### Trade off between accuracy, stability, and reconstruction

**Figure 3.**
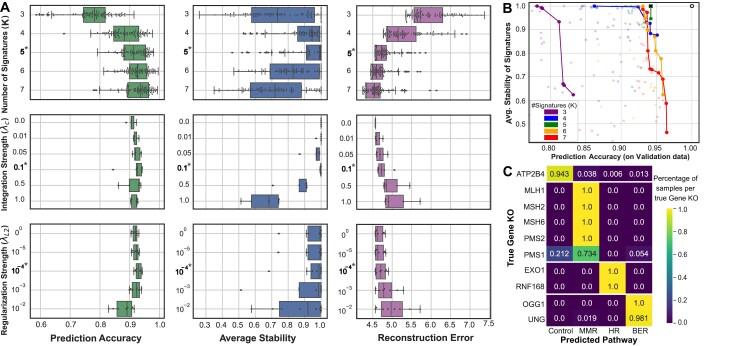
Hyperparameter optimization and prediction performance of SNMF. (**A**) Boxplots of hyperparameter optimization results using cross-validation. Each dot denotes the average evaluation metric (left: accuracy; middle: stability; bottom: reconstruction) of three validation folds per hyperparameter setting: (top) number of signatures $K$; (middle row) integration $\lambda _{C}$ (with $K = 5$); and (bottom) regularization $\lambda _{L2}$ (with $K = 5$). Final settings indicated in bold ($\ast$) (**B**) Optimal $\lambda _{C}$ and $\lambda _{L2}$ based on accuracy and stability per number of signatures $K$. Each dot denotes an average over three validation folds for one hyperparameter setting. Lines indicate pareto optimal $\lambda _{C}$ and $\lambda _{L2}$ per $K$ ($\boldsymbol{\times }$ final optimal setting; $\boldsymbol{\circ }$ theoretical perfect setting). (**C**) Confusion matrix of SNMF predictions per gene KO. Coloured and annotated by percentage of samples per gene KO (real and bootstrapped) predicted to each pathway.

The number of signatures $K$ had the largest effect on accuracy, stability, and reconstruction of SNMF (Fig. 3A and Supplementary Fig. S6). Increasing $K$ from 3 to 7 improved median accuracy (0.78 versus 0.93) and reconstruction error (5.74 versus 4.57). This was expected, since using more signatures increases model complexity and could result in a better fit to the data. This effect was less pronounced above 5 signatures. The median stability also improved when increasing $K$ from 3 to 5 signatures (0.67 versus 0.99), but decreased for $K> 5$ (0.72 for $K = 7$). This was apparent in the Pareto plot (Fig. 3B, green versus red), where extracting 7 signatures with optimal integration and regularization strengths resulted in higher accuracy compared to 5 signatures (0.964 versus 0.942), at the cost of stability (0.46 versus 1.0) (Supplementary Figs S7 and S8). The reason was that, for $K = 7$ signatures, SNMF occasionally found a signature specific to *PMS1* gene KOs (Fig. 3B, red; Supplementary Fig. S9), but not consistently across runs. We finally settled on 5 signatures ($K = 5$), since it resulted in the most reproducible signatures.

We looked into the impact of integration and regularization strengths using $K=5$ signatures. Higher integration strength improved prediction accuracy, from median 0.906 with no integration ($\lambda _c = 0.0$) to median 0.931 with integration ($\lambda _c = 0.5$), with high stability. However, further increasing the integration strength deteriorated both stability and reconstruction error, owing to a dominance of prediction over signature identification. Using a small regularization strength slightly increased the median accuracy from 0.920 without regularization to 0.938 with $\lambda _{L2} = 0.0001$. Too strong regularization led to a decrease in accuracy (median 0.899 for $\lambda _{L2} = 0.01$), and worsened stability and reconstruction error. This effect highlights the integrated nature of the SNMF model, where the direct regularization of classifier weights also indirectly affects signature learning.

We selected the final hyperparameter values based on Pareto optimality of accuracy and stability obtained for the validation data (Fig. 3B). The optimal settings form a front per $K$, where the best is closest to the theoretically perfect model with accuracy and stability 1.0 (Fig. 3B, black $\circ$). For $K = 5$, the optimal setting yielded integration $\lambda _c = 0.1$ and regularization $\lambda _{L2} = 0.0001$ (Fig. 3A and B, black $\times$), with accuracy 0.942 and stability 1.0. These settings were used to train the final SNMF model.

### Repair deficiencies are accurately predicted in cell lines

**Figure 4.**
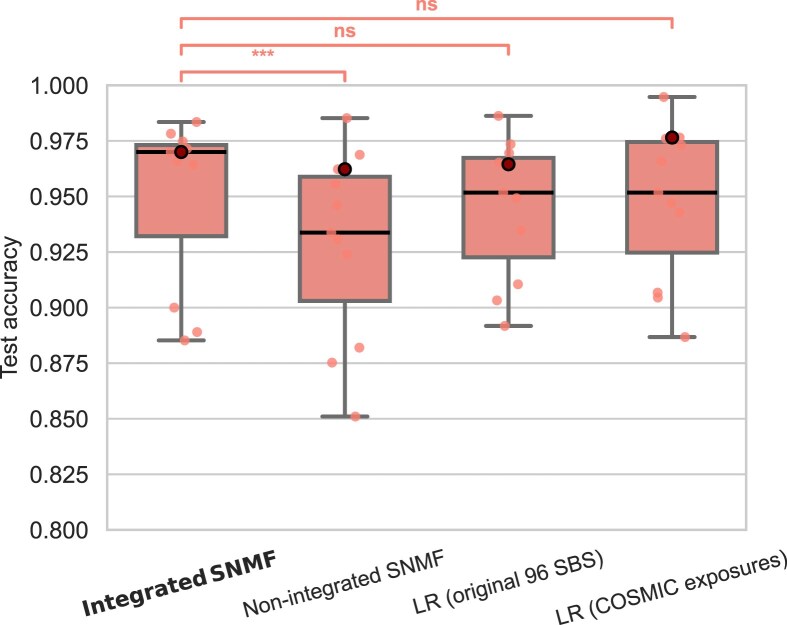
Prediction accuracy of SNMF and baseline models. Pathway prediction accuracy for 11 train-test splits of the cell line data. Significance bars indicate one-sided Wilcoxon signed-rank test results (*** $p< 0.001$, ns: not significant or $p\ge 0.05$).

One key aim of SNMF is to capture more complete and representative signatures with improved interpretability, since in principle it should be possible to build well-performing prediction models directly from the mutation profiles. Nevertheless, the signatures extracted by SNMF can only be meaningful if the model has high prediction performance as well. We confirmed that the integrated SNMF achieved high accuracy on the cell line test data (median = 0.970), which was significantly higher than the non-integrated SNMF (0.934, $p=9.8\times 10^{-4}$) (Fig. 4). Both logistic regression baselines reached similar performance levels (original 96 SBS features: 0.953; COSMIC exposures: 0.952; $p> 0.18$). This was accompanied by good signature decomposition performance, comparable to unsupervised NMF (stability: 0.97 versus 1.00; reconstruction: 4.63 versus 4.42). Given the already high baseline performance on the cell line data, prediction differences between methods were overall marginal. As a sensitivity analysis, we repeated the SNMF and non-integrated NMF comparison using ATP2B4-background corrected mutation profiles (Supplementary Fig. S10). Results showed the same qualitative pattern, with integrated SNMF retaining a modest advantage over non-integrated NMF in test accuracy (median accuracy 0.769 versus 0.746), while reconstruction performance remained comparable between settings, indicating that the observed benefit of supervision was robust to this alternative preprocessing choice.

For integrated SNMF, most incorrect predictions were made between control and MMRd samples (Fig. 3 C), where 21.2% of the *PMS1* gene KO samples were misclassified as control (accuracy 0.739). This was in line with the clustering of *PMS1* KO samples closer to control samples than to other MMRd gene KOs (Fig. 2). The remaining MMRd and HRd gene KO samples were predicted perfectly (1.0), and accuracy was also high for BERd (>0.98). For both integrated and non-integrated SNMF, all real samples were predicted correctly, with misclassifications observed only for bootstrapped samples. The non-integrated SNMF showed a higher proportion of misclassifications between *PMS1* and control samples, but all remaining gene KOs were predicted with high accuracy (Supplementary Fig. S11). Finally, the logistic regression model mainly misclassified control samples as BERd, while most PMS1 samples were predicted correctly (0.94).

Beyond repair pathway deficiencies, we also briefly explored the application of SNMF to learn models for prediction of gene-specific deficiencies and, on a completely different domain, prediction of hand-written digits using the well-known MNIST dataset. Although limited by the small sample size (1−2 samples per gene), an SNMF model trained on the cell line data using gene-specific labels identified 9 gene-level signatures and accurately predicted gene deficiency labels (0.81) (Supplementary Figs S12 and S13). On the MNIST digit prediction task, SNMF again outperformed the non-integrated method, indicating that the observed performance improvement is not confined to a single dataset or domain (Supplementary Fig. S14). Together, these findings highlight the potential of SNMF as a general framework for interpretable, task-driven representation learning beyond the context of this study.

### SNMF identifies BER subpathway deficiencies

**Figure 5.**
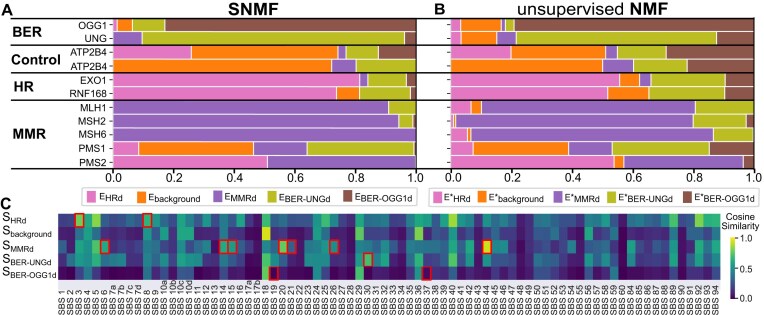
Cell line signatures identified by SNMF and NMF. (**A** and **B**) Signature exposures found by (A) SNMF and (B) NMF for the real test cell line samples (no bootstrapped samples). (**C**) Heatmap showing similarity between cell line signatures found by SNMF and COSMIC signatures. Red squares highlight COSMIC signatures with etiology related to each DNA repair pathway deficiency.

The SNMF model not only predicted all BERd samples with high accuracy but also identified two signatures clearly distinguishing the *OGG1* and *UNG* gene knockouts (Fig. 5A, brown and green, respectively). *OGG1* and *UNG* both encode for DNA glycosylase enzymes, but repair different types of DNA damage. UNG recognizes and excises uracil (deaminated bases), and its deficiency leads to C$> $T mutations when the DNA is replicated; whereas OGG1 recognizes and removes 8-oxoG (oxidized bases) and its deficiency results in G$> $T (or C$> $A) mutations (Supplementary Fig. S15) [5]. We note that individual components of these BER-associated profiles, particularly C$> $A mutations, may also reflect shared experimental background processes in the knockout screen (Supplementary Fig. S3). Since these effects are non-trivial to disentangle from a genuine role and impact of genes in the repair of oxidative damage, the BER–UNGd signature should be interpreted as the profile associated with UNG knockout under the experimental conditions of the original study, rather than as the isolated biochemical effect of UNG deficiency alone. Ultimately, the ability to recognize subpathways within a given deficiency class is a key advantage of an NMF-based model over the direct logistic regression or any other prediction-focused model that does not aim to capture underlying mutational patterns.

### SNMF signatures distinguish repair deficiencies

We compared unsupervised NMF and integrated SNMF signatures after pairing them using Hungarian matching to maximize cosine similarity. Overall, the signatures identified by both methods were similar, with mean cosine signature similarity 0.925 (Supplementary Figs S15 and S16A), and also exhibited similar exposures across the cell lines (Supplementary Fig. S16B, mean exposure Pearson’s correlation: 0.95).

The SNMF model mainly used one signature to decompose each sample, with an average of the largest exposure per sample of 0.80 (Fig. 5A). In contrast, unsupervised NMF led to more mixed exposures to additional signatures, with a largest exposure of only 0.61 on average. In this context, one could be led to interpret the contribution of multiple signatures as an indication that cells have multiple repair deficiencies instead of one, such as suggested by the exposure of MMRd samples to the BER–UNG signature (Fig. 5B, green). The exposures of unrelated signatures were reduced using integrated SNMF compared to the NMF model, suggesting that each repair deficiency was better captured into one signature by exploiting the information contained in the class labels. The unsupervised NMF tended to isolate a mutation type into one signature and to exclude it from other signatures. This effect was reduced using integrated SNMF, where multiple signatures included similar characteristic mutations (Supplementary Fig. S15).

### Cell line signatures recapitulate cancer signatures

We analysed the cell line signatures found by integrated SNMF against cancer COSMIC signatures linked to the same repair deficiency (Fig. 5C). Of all COSMIC signatures, only SBS3 has been annotated with HRd. Others have also suggested that SBS8 could be linked to HRd, and used exposures of SBS8 and SBS3 as predictors in the classifier HRDetect [4]. The HRd signature showed high similarity to SBS3 (cossim: 0.78) and SBS8 (cossim: 0.65). We note that EXO1 and RNF168 are not canonical HR genes, and therefore the HRd signature could be more broadly interpreted here as being related to deficiency in double-strand break repair. For MMRd, COSMIC lists seven partially overlapping signatures [19], which have been reduced to two main processes [20]. One is characterized by T$> $C mutations, with high similarity to SBS21 and SBS26, and relates to *PMS2*-based MMRd; the other contains C$> $T mutations, has high similarity to SBS6, SBS15, and SBS44 and relates to *MLH1/MSH2/MSH6*-based MMRd (Supplementary Fig. S15) [21]. The MMRd signature found by SNMF was most similar to the latter (SBS44), explaining why *PMS1/2* were not captured as well as other relevant gene KOs (Supplementary Fig. S17). For BERd, two COSMIC signatures relate to *OGG1* deficiency: SBS18, annotated with DNA damage by reactive oxygen; and SBS36, linked to *MUTYH* deficiency. The BER–OGG1d signatures found by SNMF showed high similarity to SBS18 (cossim: 0.76) and, to a lesser extent, SBS36 (cossim: 0.52). The BER–UNGd was most similar to SBS30 (cossim: 0.89), related to deficiency of *NTHL1*, another glycosylase that recognizes abnormal pyrimidines similarly to *UNG* [5].

### Transfer of cell line signatures to tumours reveals associations with mutations in DDR genes

**Figure 6.**
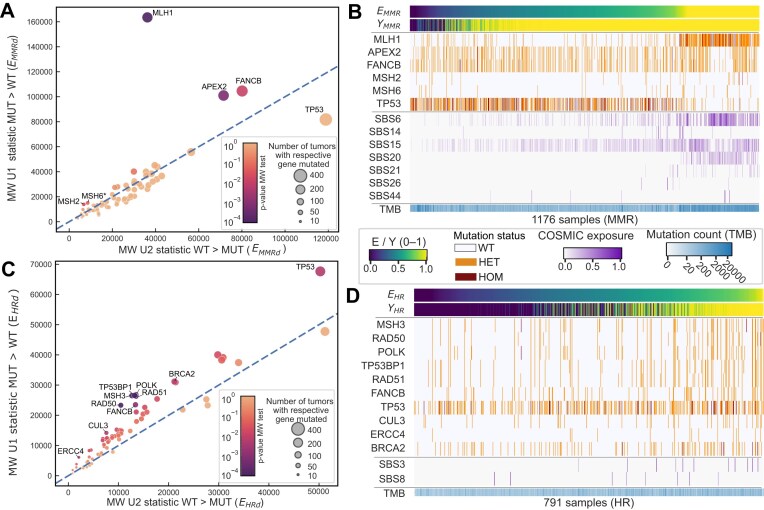
Integrated SNMF predictions on TCGA tumour data, and genes related to the MMR and HR repair deficiencies. (Top, **A** and **B**) MMRd in COAD, STAD, and UCEC tumours. (Bottom, **C** and **D**) HRd in BRCA tumours. Left: Mann–Whitney *U* test statistics per repair gene, comparing the signature exposure for wild-type tumours (U2) against tumours with the mutated gene (U1). In panel (A), labelled genes have $p < 0.05$ (with MSH6* $p < 0.10$), and in panel (C), labelled genes have $p < 0.01$. Right: Top two rows represent the exposure ($E$) and prediction probability ($Y$) found by SNMF. The remaining rows represent mutation status of tumour samples for the genes with the lowest MWU *P*-value. For panels (B) and (D), we additionally show the exposure of the related COSMIC signatures and the total SBS mutation count (TMB, on log$_{10}$ scale). (B: MMRd genes with $p \le 0.05$; MSH6* with $p < 0.10$ is additionally shown, and *TP53* is included to illustrate the negative correlation with $E_{MMRd}$. D: HRd genes with $p < 0.01$).

We evaluated the potential to transfer SNMF-learned cell line signatures and model to tumours. We focused on HRd and MMRd samples, as these deficiencies are better characterized in the literature, including some well-known associations with mutation status of specific genes (Supplementary Figs S18 and S19). We did not analyse BERd further, since only two samples carried homozygous mutations in a BER gene (Supplementary Fig. S20), and BERd-related COSMIC signatures (SBS18/30) showed comparatively low exposure in the TCGA cohort (Supplementary Figs S21 and S22). All tumours with a homozygous mutated MMR gene were predicted as MMRd (recall 1.0), most carrying *MLH1* mutations ($30/37$, Supplementary Table S2A). Additionally, eleven of the twelve tumours yielding a homozygous mutated HR gene were predicted as HRd (recall 0.92), where the misclassified tumour was BRCA1 deficient (Supplementary Table S2B). Overall, the SNMF model was more sensitive to the exposure of the MMRd signature for MMRd prediction than to that of the HRd signature for HRd prediction (Fig. 6B and D, and Supplementary Fig. S23).

We further analysed tumour exposures to SNMF cell line signatures and mutation status of DDR genes to identify potential associations (Fig. 6A and C). For the MMRd related cancer types, mutations in five genes (*MLH1, APEX2, FANCB, MSH2, MSH6*) significantly associated with exposure to the MMRd signature ($E_{MMRd}$; Fig. 6A and Supplementary Fig. S24, $p< 0.10$ for *MSH6*, others $p< 0.05$). Mutations in the *MLH1* gene had a strong effect on $E_{MMRd}$, confirming the known role of MLH1 in MMR [22]. The effect for APEX2 and FANCB was no longer significant after excluding tumours with co-occurring mutations in other MMR genes (Supplementary Fig. S25), including MLH1, and we did not find literature linking APEX2 or FANCB to MMR. The MMR genes *MSH2* and *MSH6*, mutated in 28 and 32 tumours, were initially associated with $E_{MMRd}$ as well ($p < 0.05$). After excluding tumours with mutations in other MMR genes, also too few samples remained (9 and 6) to test the effect. Interestingly, tumours with high $E_{MMRd}$ were depleted of *TP53* mutations (Fig. 6A and B).

For HRd, 24 genes yielded an association with $E_{HRd}$ ($p < 0.05$), of which 10 were more strongly significant ($p < 0.01$) and 8 still showed an effect after excluding tumours with mutations in other HR genes (Fig. 6C and D, and Supplementary Figs S25 and S26). Among this latter group were three core HR genes (*TP53BP1, BRCA2, RAD51*), two genes related to HR (*POLK, TP53*), and two genes linked to other processes (*MSH3, FANCB*). The link of FANCB with HRd supports the known interplay between the Fanconi anaemia (FA) and HR pathways, as well as the role of FANCB in the FA–BRCA pathway [23]. Other HR genes linked to increased risk of cancer, such as *BRCA1, PALB2, BARD1, BRIP1*, or *ATM* [24], did not fall below the more stringent threshold $p< 0.01$. Notably, the first three had $p< 0.05$. It has been suggested that there could be two types of HRd, *BRCA1*–HRd and *BRCA2*–HRd, resulting in distinct mutational patterns [25]. A potential explanation for the smaller effect of *BRCA1* and *BRCA1*-related genes could be that the HRd signature found by SNMF ($S_{HRd}$) was less specific for *BRCA1*–HRd.

To assess whether these associations identified between DDR genes and tumour exposures to SNMF-learned signatures depended on supervision, we repeated the analysis using the matched non-integrated NMF signatures learned from the same cell line data ($\lambda _c=0$; Supplementary Figs S27–S30). For MMRd, the integrated SNMF and non-integrated NMF tumour exposures were highly concordant (Pearson’s $r=0.97$) and recovered the same significant DDR gene associations (*MLH1, APEX2, MSH2, FANCB*, and *MSH6*; Supplementary Fig. S27). Thus, based on the rather strong and well-delineated signal generated by the gene knockouts in cell lines, non-integrated NMF could already capture the signature of the underlying processes. For HRd, however, the NMF HRd-associated signature did not recover significant associations with HR-related genes at the same threshold (Supplementary Fig. S28), whereas the NMF control/background signature was associated with a subset of the genes identified using the SNMF HRd exposure, including *RAD50, MSH3, POLK, TP53BP1, FANCB*, and *RAD51* (Supplementary Figs S29 and S30). This suggests that the non-integrated NMF model captured related HRd variation, but seemed to assign it to the signature that was most similar to background/control. In this setting, supervision appeared to better preserve the intended alignment between the cell line perturbation label and confirmed gene knockout etiology, the learned HRd signature, and the downstream association of tumour exposures to the signature with relevant HR-related genes. More broadly, this also highlights the opportunity for perturbation-derived signatures as a complement to tumour-derived catalogues, with cell line screens providing confirmed etiological anchors and tumour-derived references capturing a wider range of operative mutational processes.

To ensure the soundness of our association analysis based on Mann–Whitney (MW) *U* tests, we verified that the number of mutated DDR genes did not show an obvious association with total mutation count (Supplementary Fig. S31). We controlled directly for total tumour mutation burden using analysis of covariance (ANCOVA), modelling signature exposure as a function of gene mutation status with total SBS mutation count as a covariate. The ANCOVA results were consistent with the original MW analysis, showing that the identified associations were robust and not driven primarily by mutation burden (Supplementary Figs S32 and S33).

We note that the exposures we obtained by fitting the limited set of SNMF-learned cell line signatures to tumours could differ in absolute terms from those in published studies, given that the latter are identified *de novo* or fitted across the broader range of tumour-derived signatures, many of which may not be represented in the cell line reference data. For this reason, we clarify that such exposures cannot be interpreted as directly quantifying the prevalence of the associated processes in tumours. Since our goal was to assess the potential of SNMF-learned signatures to capture relevant processes in tumours, rather than to quantify prevalence, we fit the SNMF signatures as-is and analyse the respective exposures only in relative terms: when testing for association with DDR genes, we seek to determine if exposures are relatively larger on average in tumours with versus tumours without mutations in a given DDR gene. We confirmed that tumour exposures to SNMF-learned cell line signatures correlated with tumour exposures to COSMIC signatures (Spearman correlations: MMRd $\rho \approx 0.703$ with $p\approx 3.7 \times 10^{-146}$, HRd $\rho \approx 0.142$ with $p\approx 6.4 \times 10^{-5}$; Supplementary Fig. S34). The fitting of cell line signatures assigned non-zero exposures more often compared to the fitting of COSMIC signatures for both MMRd and HRd. This is unsurprising and likely stems from redistribution of exposures to tumour-related processes not represented in the cell line signatures. Such redistribution affects absolute values, whereas relative consistent trends driven by associations with specific genes should be preserved, as confirmed by our results (Fig. 6). Though we do not see it here, we caution that if exposures to a mutational process not represented in the reference signature set were to be systematically assigned to one specific reference signature, one could potentially see associations emerge with unexpected genes. Therefore, our analysis is merely confirmatory against established knowledge rather than aimed at making new discoveries. More generally, signature fitting is challenging, and even using the full range of COSMIC signatures is not guaranteed to produce exposures in line with published studies. For instance, fitting COSMIC signatures to the TCGA tumours led to sparse assignment of MMRd-related signature SBS44 for COAD, STAD, and UCEC tumours (Fig. 6B), whereas SBS44 is the most prevalent MMRd signature for those cancer types across the Signal and COSMIC databases [26, 27]. Likewise, we saw little assignment of HRd-related COSMIC signatures SBS3 and SBS8 (Fig. 6D). While differences could be cohort-specific, another plausible explanation is that exposures might be underestimated due to challenges of concurrently fitting similar signatures (e.g. 7 COSMIC MMRd-related SBS6/14/15/20/21/26/44) or “flat” signatures (e.g. COSMIC SBS3 and SBS5), which could redistribute exposures among such signatures. In effect, and in contrast with exposures to the SNMF-derived HRd cell line signatures (Fig. 6C), exposures to COSMIC HRd signatures SBS3/SBS8 did not show significant associations with any DDR genes. Together, these results confirm that exposures assigned to any given signature also strongly depend on the complete set of signatures being fitted. They further illustrate a general trade off of fitting catalogues of mutational signatures to tumours: while fitting restricted signature sets can overassign exposures to available signatures, fitting comprehensive catalogues such as COSMIC can underestimate exposures by distributing them across similar or partially redundant signatures. Finally, we note that SNMF-learned cell line signatures can be alternatively fitted together with non-redundant tumour-derived signatures to obtain exposures across the full range of processes operative in tumours, if desired. We expect such combined fitting to yield a more complete reconstruction of the mutational processes in tumours (Supplementary Figs S34 and S35), while retaining etiological anchors provided by the gene perturbation-guided signatures, though exposure estimates will remain dependent on the chosen reference set.

## Conclusion

We explored a SNMF approach to integrate mutational signature learning with multiclass DDRd prediction, with the aim to identify signatures characteristic of different types of DDR deficiencies based on disruption to pathways or individual genes.

The SNMF model achieved high accuracy for the relatively easy task of predicting HR, BER, and MMR deficiency in hiPS cell lines with relevant gene KOs, on par with prediction models without signature identification. Likewise, SNMF preserved the high signature stability and low reconstruction error of independent signature learning, which confirmed the ability of SNMF to jointly optimize both objectives. The number of signatures had the most impact on SNMF performance, and larger integration strength improved accuracy at the cost of signature stability. Improvement over unsupervised NMF in the cell line data was small, yet consistent and statistically significant. This confirmed that much of the discriminative structure of the data could already be captured by unsupervised decomposition, owing to the robust experimental design with well-isolated effects of individual gene knockouts. Nevertheless, SNMF enabled further refinement guided by data labels, enhancing predictive performance and offering direct etiological interpretation. Attesting to the value of integrating signature learning with DDRd prediction, the SNMF-identified signatures provided a more complete representation of each repair deficiency class, whereas signature learning on its own (via unsupervised NMF) tended to explain most deficiencies using multiple signatures. The (S)NMF model further captured cell line signatures linked to distinct subpathways, such as OGG1 and UNG in BER, highlighting the interpretability provided by the SNMF integrated signature learning over direct prediction from mutational profiles, and thereby its potential to enhance our understanding of DDR.

On transfer to tumours, the cell line SNMF model recapitulated tumour-related COSMIC signatures and identified HR and MMR deficiencies in TCGA tumours with high recall, although prediction was too sensitive to MMR signature exposure. These results are encouraging given the limited scope of currently available knockout data, but also highlight the need for future perturbation screens that explicitly consider and disentangle genuine biological effects from experimental background processes. Larger DDRd screens with matched controls, sufficient replication, and complementary induced DNA damage conditions could help separate culture-associated damage from induced damage and repair-deficiency effects, enabling the learning of signatures with clearer etiological interpretation. Combined with model adaptations that better bridge between cell lines and tumours, such as rejection options, semi-supervised learning, or multi-label classification for co-occurring deficiencies, perturbation-derived models could support more systematic and complementary annotation of DDR-associated mutational processes in tumours.

Notably, exposure of tumours to SNMF-learned cell line signatures also revealed associations with mutation status of relevant DDR genes, such as MLH1 for MMRd and BRCA2 for double-strand break repair and HR deficiencies. Further gene-level analysis of the SNMF repair pathway deficiency prediction model suggested that deficiency in genes linked to a given pathway could lead to distinct mutation patterns, such as BRCA1- versus BRCA2-HRd, or MLH1- versus PMS2-MMRd, highlighting the importance of discerning subtype repair deficiencies. With conventional unsupervised NMF, granularity can be explored by varying the number of signatures. However, optimizing stability and reconstruction does not guarantee the biological relevance of the identified signatures, and using known labels only for post-hoc annotation can be a missed opportunity. The SNMF model directly leverages pathway or gene labels during learning to guide the signature identification, enabling flexible and fine-grained characterization of mutational patterns at multiple levels of granularity. Beyond the benefits of supervised signature learning for DDRd prediction using SNMF, we showed the potential of cell line SNMF signatures to unravel repair deficiencies in patient tumours. The results are especially encouraging considering the limited data available to train the model, which is nevertheless quite unique and one of the most comprehensive datasets of this kind. We envision the use of SNMF-like models to learn patterns from high-throughput cell line experiments with induced DDR deficiencies to decipher signatures in patient tumours and generate clinically relevant insights aimed at improving precision cancer therapy.

## Supplementary Material

lqag078_Supplemental_File

## Data Availability

All data used in this study are publicly available. CRISPR knockout cell line mutation calls are available at https://doi.org/10.17632/ymn3ykkmyx. COSMIC mutational signatures (v3.2) can be accessed from https://cancer.sanger.ac.uk/signatures/. Whole-exome MC3 somatic mutation calls from The Cancer Genome Atlas (TCGA) are available as described in [14]. SNMF is available at https://github.com/joanagoncalveslab/SNMF.
