# Supplementary material for: Etiology-guided mutational signature learning from DNA repair knockouts in cell lines using supervised NMF
Source: NAR Genom Bioinform. 2026 Aug 6;8(3):lqag078. doi: 10.1093/nargab/lqag078 (PMC13443540; doi:10.1093/nargab/lqag078)
Supplement: Supplementary file 1 [file lqag078_supplemental_file.pdf]

Supplementary Material

A. Extended Methods: derivation of the update rules and derivatives for the SNMF optimization algorithm

A.1. Matrix definitions

$\boldsymbol{X}_{N,T}$: Input matrix of (normalized) frequencies of T mutation types for each of the N samples (mutational profiles)

$\boldsymbol{Y}_{N,O}$: Output class label matrix, with O class labels for each of the N samples (one-hot encoded DNA repair deficiencies)

$\boldsymbol{S}_{K,T}$: Mutational signature matrix, denoting the frequencies of T mutation types for each of the K signatures

$\boldsymbol{E}_{N,K}$: Exposure matrix, containing the contribution of each of the K signatures to each of the N samples

$\boldsymbol{W}_{K,O}$: Coefficients of logistic regression, weighing contribution of each of the K exposures to prediction of each of the O classes

with:

N : number of samples (and mutational profiles)

T : number of mutation types

K : number of signatures

O : number of output classes, corresponding to DNA repair pathway deficiencies and control

A.2. Loss function and update rules for optimization by gradient descent

The loss function $\mathcal{L}_{tot}$ optimized by S-NMF combines a reconstruction loss $\mathcal{L}_r$ with a classification loss $\mathcal{L}_c$ weighed by a factor λ_c as follows.

$$\mathcal{L}_{tot} = \mathcal{L}_r + \lambda_c \mathcal{L}_c$$

The reconstruction loss $\mathcal{L}_r$ is defined as the Frobenius reconstruction error of the decomposition of matrix $\boldsymbol{X}$ into matrices $\boldsymbol{E}$ and $\boldsymbol{S}$.

$$\mathcal{L}_r = ||\boldsymbol{X} - \boldsymbol{ES}||_F^2$$

The classification loss $\mathcal{L}_c$ is the categorical cross-entropy loss:

$$\mathcal{L}_c = -\sum_{n=1}^{N}\sum_{o=1}^{O} y_{n,o} \log\left(\hat{y}_{n,o}\right),$$

where n and o are sample and output class indices, respectively, $y_{n,o}$ is the true class label (one-hot encoded), and $\hat{y}_{n,o}$ is the predicted soft class label calculated as follows.

$$\hat{y}_{n,o} = softmax\left(\boldsymbol{E_{n,*}W_{*,o}}\right) = \frac{e^{\boldsymbol{E}_{n,*}\boldsymbol{W}_{*,o}}}{\sum_{p=1}^{P} e^{\boldsymbol{E}_{n,*}\boldsymbol{W}_{*,p}}}$$

The loss function $\mathcal{L}_{tot}$ is optimized using gradient descent, following the iterative updates below.

$$\boldsymbol{S} \leftarrow \boldsymbol{S} - \eta_S \cdot \nabla_{\boldsymbol{S}}\mathcal{L}_{tot}$$
$$\boldsymbol{E} \leftarrow \boldsymbol{E} - \eta_E \cdot \nabla_{\boldsymbol{E}}\mathcal{L}_{tot}$$
$$\boldsymbol{W} \leftarrow \boldsymbol{W} - \eta_W \cdot \nabla_{\boldsymbol{W}}\mathcal{L}_{tot}$$

Symbols η denote the respective learning rates (see A.7), and ∇ the (partial-) derivatives of the total loss. In the next sections, we work out the derivatives for the update rules with respect to the reconstruction and the classification losses.

A.3. Derivatives of the reconstruction loss:

Firstly, we calculate the derivative with respect to the reconstruction loss. This is identical to what existing methods that applied gradient descent to optimize NMF (15). For completeness, we will show the calculation.

Firstly, the frobenius reconstruction loss can be rewritten into four trace terms.

$$\begin{aligned}
||\boldsymbol{X} - \boldsymbol{E}\boldsymbol{S}||_F^2 &= Tr((\boldsymbol{X}^T - \boldsymbol{S}^T\boldsymbol{E}^T)(\boldsymbol{X} - \boldsymbol{E}\boldsymbol{S})) \\
&= Tr(\boldsymbol{X}^T\boldsymbol{X}) - Tr(\boldsymbol{X}^T\boldsymbol{E}\boldsymbol{S}) - Tr(\boldsymbol{S}^T\boldsymbol{E}^T\boldsymbol{X}) + Tr(\boldsymbol{S}^T\boldsymbol{E}^T\boldsymbol{E}\boldsymbol{S}) \\
since, & \\
||\boldsymbol{X}||_F^2 &= Tr(\boldsymbol{X}^T\boldsymbol{X}) \\
Tr(A+B) &= Tr(A) + Tr(B)
\end{aligned}$$

We take the derivative of each of the four trace terms in the loss function. Shown are the derivatives with respect to the exposure matrix $\boldsymbol{E}$, a similar procedure is followed to obtain derivatives with respect to $\boldsymbol{S}$ (applied mathematical rules between parentheses):

$$\begin{aligned}
\nabla_S Tr(\boldsymbol{X}^T\boldsymbol{X}) &= 0 && - \\
\nabla_S Tr(\boldsymbol{X}^T\boldsymbol{E}\boldsymbol{S}) &= \boldsymbol{E}^T\boldsymbol{X} && (\nabla_x Tr(\boldsymbol{A}\boldsymbol{X}) = \boldsymbol{A}^T) \\
\nabla_S Tr(\boldsymbol{E}^T\boldsymbol{S}^T\boldsymbol{X}) &= \boldsymbol{E}^T\boldsymbol{X} && (\nabla_x Tr(\boldsymbol{X}^T\boldsymbol{A}) = \boldsymbol{A}) \\
\nabla_S Tr(\boldsymbol{S}^T\boldsymbol{E}^T\boldsymbol{E}\boldsymbol{S}) &= ((\boldsymbol{E}^T\boldsymbol{E}) + (\boldsymbol{E}^T\boldsymbol{E})^T)\boldsymbol{S} = 2\boldsymbol{E}^T\boldsymbol{E}\boldsymbol{S} && (\nabla_x Tr(\boldsymbol{X}^T\boldsymbol{A}\boldsymbol{X}) = (\boldsymbol{A} + \boldsymbol{A}^T)\boldsymbol{X})
\end{aligned}$$

The final derivatives $\nabla_{\boldsymbol{E}}\mathcal{L}_r$ and $\nabla_{\boldsymbol{S}}\mathcal{L}_r$ of the reconstruction loss $\mathcal{L}_r$ with respect to $\boldsymbol{E}$ and $\boldsymbol{S}$ are as follows.

$$\nabla_{\boldsymbol{S}}\mathcal{L}_r = -2\boldsymbol{E}^T\boldsymbol{X} + 2\boldsymbol{E}^T\boldsymbol{E}\boldsymbol{S} \quad (1)$$

$$\nabla_{\boldsymbol{E}}\mathcal{L}_r = -2\boldsymbol{X}\boldsymbol{S}^T + 2\boldsymbol{E}\boldsymbol{S}\boldsymbol{S}^T \quad (2)$$

A.4. Derivatives of the classification loss:

Next, we calculate the gradients of classification component of the total loss. The main difference with a regular logistic regression is that in our case we not only apply gradient descent on the classification weights W but also the exposures E which could considered the input data in a regular logistic regression.

Here we focus on the categorical cross-entropy term and in section A.5 the gradient of the L2-regularization term will be calculated.

$$\mathcal{L}_c = -\sum_{n=1}^{N}\sum_{o=1}^{O} y_{n,o}\log(\hat{y}_{n,o}) + \lambda_{L2}\sum_{w\in\boldsymbol{W}} w^2 \tag{3}$$

For a more convenient calculation of the derivatives, we define $\boldsymbol{Z}$ as the product of the exposures and weights (eq. 5). $\boldsymbol{Z}$ can then be used as input to the softmax.

$$\text{with,} \qquad \hat{y}_{n,o} = softmax(z_{n,o}) = \frac{e^{z_{n,o}}}{\sum_{p=1}^{P} e^{z_{n,p}}} \tag{4}$$

$$\text{with,} \qquad \boldsymbol{Z} = \boldsymbol{EW} \tag{5}$$

Having defined $\boldsymbol{Z}$, we can define the derivative of the cross-entropy loss $d\mathcal{L}_c$ (eq. 6). Taking the derivative using matrices is more complex compared to scalars since the order of the matrices is relevant. Taking this into account, the following rule can be applied:

$$d\mathcal{L}_c = \frac{\partial\mathcal{L}_c}{\partial\boldsymbol{Z}} : dZ \tag{6}$$

$$\text{with,} \qquad \boldsymbol{A} : \boldsymbol{B} = \langle\boldsymbol{A},\boldsymbol{B}\rangle_F$$

where : indicates the Frobenius inner product.

Next, both terms in the derivative of the cross entropy loss $d\mathcal{L}_c$ (eq. 6) need to be calculated.

Firstly, the partial derivative of the cross-entropy loss w.r.t. $\boldsymbol{Z}$ ($\frac{\partial\mathcal{L}_c}{\partial\boldsymbol{Z}}$, eq. 7). This partial derivative is similar to regular logistic regressions, and is as calculated as follow:

$$\frac{\partial\mathcal{L}_c}{\partial\boldsymbol{Z}} = (\hat{\boldsymbol{Y}} - \boldsymbol{Y}) \tag{7}$$

The second term in eq. 6, the full derivative of $\boldsymbol{Z}$ (dZ), can be further defined in terms of the exposures and weights:

$$\boldsymbol{Z} = \boldsymbol{EW} \tag{8}$$

$$dZ = dE\boldsymbol{W} + \boldsymbol{E}dW \tag{9}$$

Taken together, $\frac{\partial\mathcal{L}_c}{\partial\boldsymbol{Z}}$ (eq. 7) and dZ (eq. 9) can be substituted in the derivative of the cross entropy loss (eq. 6).

$$\begin{aligned} d\mathcal{L}_c &= \frac{\partial\mathcal{L}_c}{\partial\boldsymbol{Z}} : dZ \\ &= (\hat{\boldsymbol{Y}} - \boldsymbol{Y}) : (dE\boldsymbol{W} + \boldsymbol{E}dW) \\ &= (\hat{\boldsymbol{Y}} - \boldsymbol{Y}) : dE\boldsymbol{W} + (\hat{\boldsymbol{Y}} - \boldsymbol{Y}) : \boldsymbol{E}dW \\ &= (\hat{\boldsymbol{Y}} - \boldsymbol{Y})\boldsymbol{W}^T : dE + \boldsymbol{E}^T(\hat{\boldsymbol{Y}} - \boldsymbol{Y}) : dW \end{aligned}$$

Finally, for the gradient w.r.t $\boldsymbol{E}$, $\boldsymbol{W}$ is constant (i.e. $dW = 0$) and for the w.r.t. $\boldsymbol{W}$, $\boldsymbol{E}$ is constant (i.e. $dE = 0$). This result in the final derivatives $\nabla_{\boldsymbol{E}}\mathcal{L}_c$ and $\nabla_{\boldsymbol{W}}\mathcal{L}_c$ of the cross-entropy loss w.r.t. $\boldsymbol{E}$ and $\boldsymbol{W}$:

$$\nabla_{\boldsymbol{W}}\mathcal{L}_c = \frac{\partial\mathcal{L}_c}{\partial\boldsymbol{W}} = \boldsymbol{E}^T(\hat{\boldsymbol{Y}} - \boldsymbol{Y}) \tag{10}$$

$$\nabla_{\boldsymbol{E}}\mathcal{L}_c = \frac{\partial\mathcal{L}_c}{\partial\boldsymbol{E}} = (\hat{\boldsymbol{Y}} - \boldsymbol{Y})\boldsymbol{W}^T \tag{11}$$

A.5. Derivative of L2-regularization term

Finally, the derivative of the L2 regularization term w.r.t. $\boldsymbol{W}$. Which is the same as standard derivations.

$$\frac{\partial(\lambda_{L2}\sum_{w\in\boldsymbol{W}} w^2)}{\partial\boldsymbol{W}} = 2\lambda_{L2}W \tag{12}$$

A.6. Final gradients

To conclude we combine the partial derivatives of individual terms in the loss function. With regard to the signatures, the derivative only contains a term from of the reconstruction loss (eq. 1). For the exposure, we have a term from both the reconstruction loss (eq. 2) and the cross-entropy loss (eq. 11). For the classifier weight, we have a term from the cross-entropy loss (eq. 10) and from the L2-regularization (eq. 12) of the individual terms in the loss function to get the final gradients of the total loss $\mathcal{L}_{tot}$.

$$\nabla_{\boldsymbol{S}}\mathcal{L}_{tot} = -2\boldsymbol{E}^T\boldsymbol{X} + 2\boldsymbol{E}^T\boldsymbol{ES} \tag{13}$$

$$\nabla_{\boldsymbol{E}}\mathcal{L}_{tot} = -2\boldsymbol{XS}^T + 2\boldsymbol{ESS}^T + \lambda_c(\hat{\boldsymbol{Y}} - \boldsymbol{Y})\boldsymbol{W}^T \tag{14}$$

$$\nabla_{\boldsymbol{W}}\mathcal{L}_{tot} = \lambda_c(\boldsymbol{E}^T(\hat{\boldsymbol{Y}} - \boldsymbol{Y}) + 2\lambda_{L2}\boldsymbol{W}) \tag{15}$$

A.7. (Adaptive) Learning rates:

We use the same adaptive learning rates η_S and η_E for optimization of $\boldsymbol{S}$ and $\boldsymbol{E}$ as in (15).

We do not want the integration strength (λ_c) to effect the optimization of the regression weights $\boldsymbol{W}$, to prevent having a derivative of zero when setting $\lambda_c = 0$. Therefor, we divide our wanted constant learning rate μ_W by λ_c. This cancels out the integration strength term (λ_c) in the derivative $\nabla_{\boldsymbol{W}}\mathcal{L}_{tot}$ (eq.15) and leads to a constant learning rate μ_W in the final update formula.

$$\eta_S = \frac{\boldsymbol{S}}{2\boldsymbol{E}^T\boldsymbol{E}\boldsymbol{S}} \tag{16}$$

$$\eta_E = \frac{\boldsymbol{E}}{2\boldsymbol{E}\boldsymbol{S}\boldsymbol{S}^T} \tag{17}$$

$$\eta_W = \frac{\mu_W}{\lambda_c} \tag{18}$$

A.8. Multiplicative update formulas.

The final multiplicative update formulas are obtained by substituting the derivatives of the loss and the learning rates in the gradient descent update formulas. ($\odot$ and division are element-wise).

$$\boldsymbol{S} \leftarrow \boldsymbol{S} \odot \frac{\boldsymbol{E}^T\boldsymbol{X}}{\boldsymbol{E}^T\boldsymbol{E}\boldsymbol{S}} \tag{19}$$

$$\boldsymbol{E} \leftarrow \boldsymbol{E} \odot \frac{\boldsymbol{X}\boldsymbol{S}^T - \frac{\lambda_c}{2}(\hat{\boldsymbol{Y}} - \boldsymbol{Y})\boldsymbol{W}^T}{\boldsymbol{E}\boldsymbol{S}\boldsymbol{S}^T} \tag{20}$$

$$\boldsymbol{W} \leftarrow \boldsymbol{W} - \mu_W(\boldsymbol{E}^T(\hat{\boldsymbol{Y}} - \boldsymbol{Y}) + 2\lambda_{L2}\boldsymbol{W}) \tag{21}$$

B. Extended Results

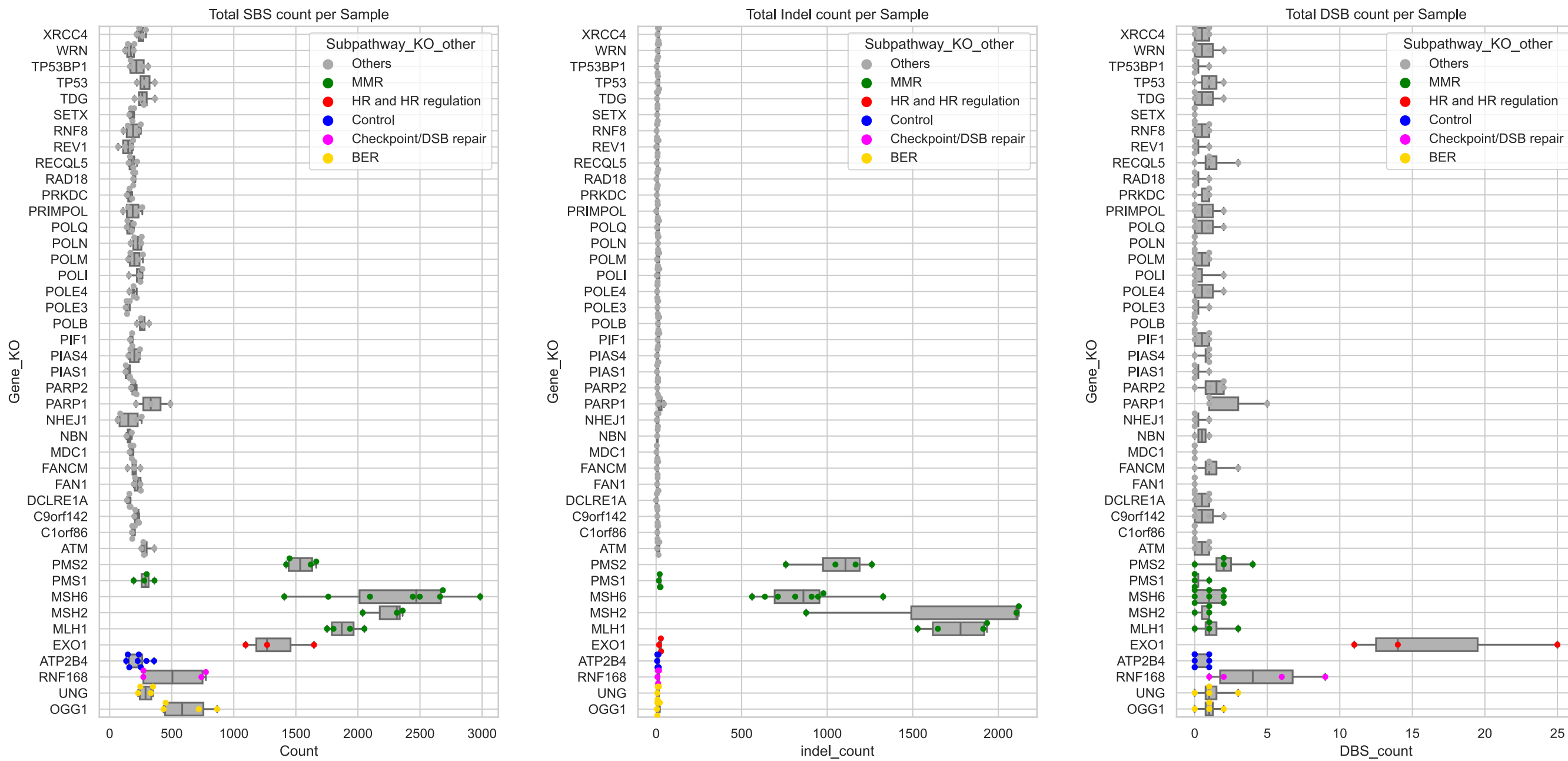

(A) Total number of single base substitutions (SBSs) per sample.

(B) Total number of small insertions and deletions (Indels) per sample.

(C) Total number of double base substitutions (DBSs) per sample.

Fig. S1: **Mutation counts per sample categorized per gene KO.** Grey are the non-distinctive gene KOs; other samples are the gene KOs used for SNMF evaluation, colored by their related repair pathway.

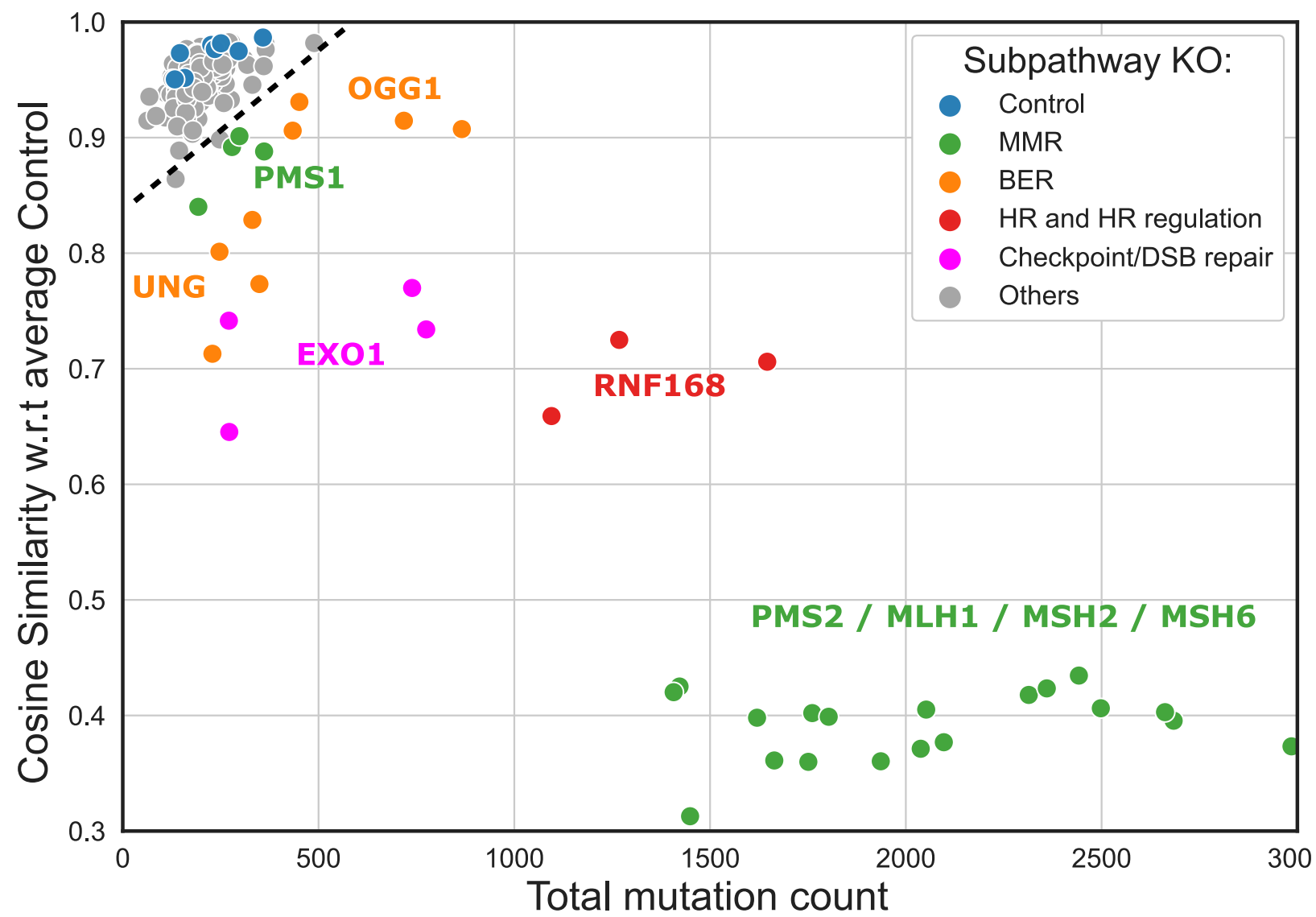

Fig. S2: **Gene knockout and bootstrapped training samples.** Total mutation count and cosine similarity with average profile of control samples, per sample (dot) annotated with gene KO and coloured by repair pathway. The dashed line indicates the threshold used to select mutation profiles: only gene KOs for which all samples yielded higher mutation counts and/or lower similarity to controls than indicated by the dashed line were selected.

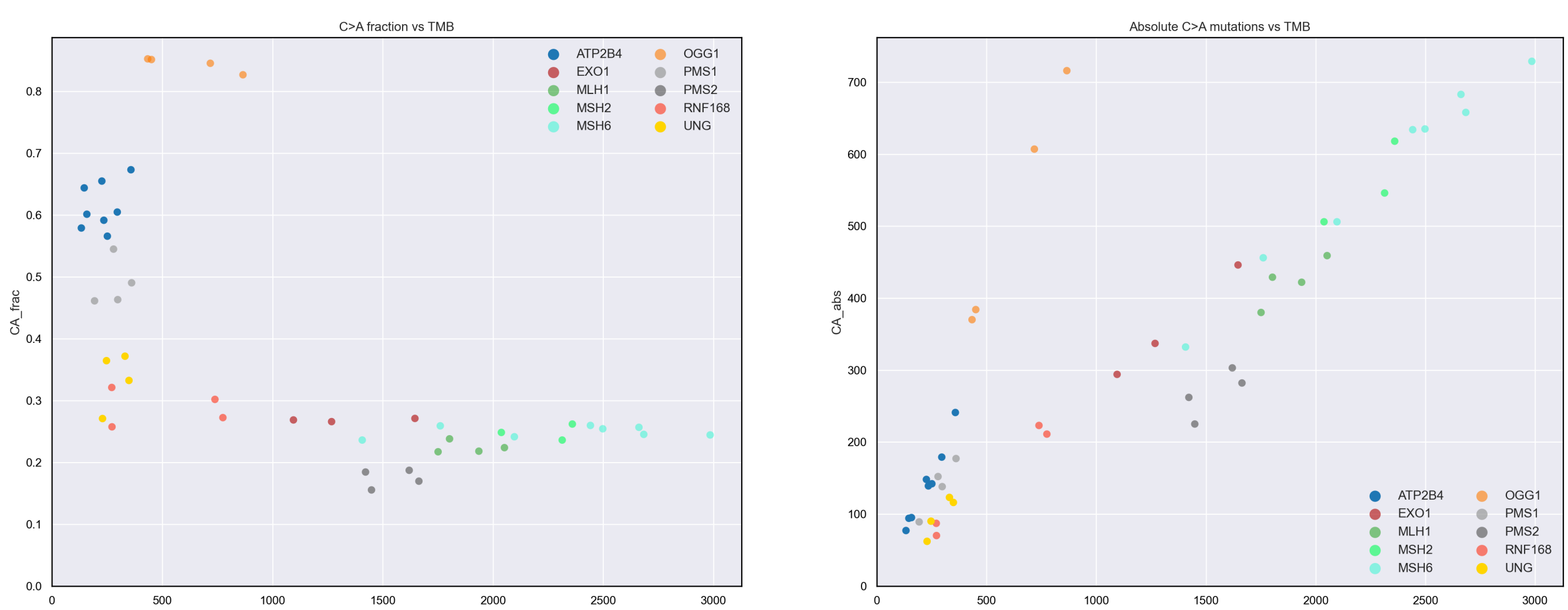

Fig. S3: **Relationship between C>A mutations and total mutation burden across gene knockouts. Left:** Fraction of C>A mutations relative to the total mutation count. **Right:** Absolute C>A mutation count. Both quantities are shown as a function of total mutation burden (TMB) across gene knockout samples, illustrating the distinction between compositional C>A fractions and absolute C>A mutation counts.

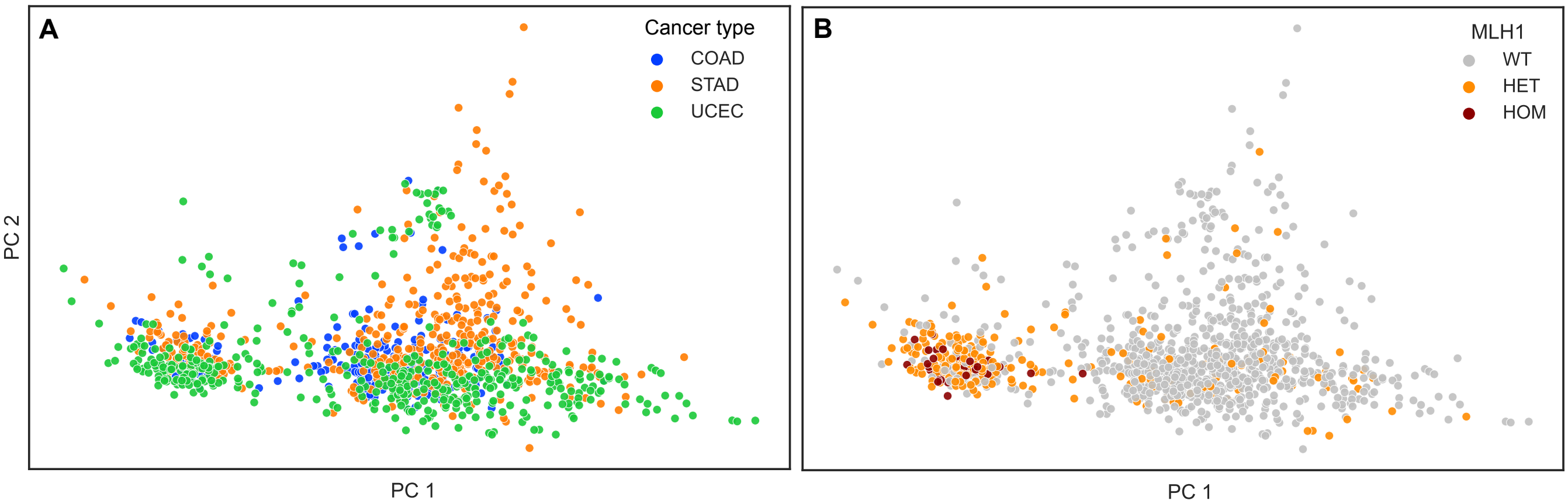

Fig. S4: **PCA of mutational profiles of patients with MMR-related cancer types colon (COAD), stomach (STAD) and uterine (UCEC).** Samples are colored by **A)** the cancer type of each tumour; **B)** the mutation status of MLH1 (WT: wild-type, HET: heterozygous-, HOM: homozygous-mutation).

Gene KO	DNA Repair pathway	Total # Samples	# Test	# Train	# Fold 1	# Fold 2	# Fold 3
ATP2B4	Control	8	2	6	2	2	2
MSH6	Mismatch Repair	8	1	7	3	2	2
MSH2		3	1	2	0	1	1
MLH1		4	1	3	1	1	1
PMS2		4	1	3	1	1	1
PMS1		4	1	3	1	1	1
EXO1	HR	3	1	2	1	0	1
RNF168		4	1	3	1	1	1
OGG1	Base Exision Repair	4	1	3	1	1	1
UNG		4	1	3	1	1	1
	Total:	46	11	35	12	11	12

Fig. S5: **Table showing the number of samples and how they are subdivided over the test set and training folds.** Shown are the Gene KOs (rows), their annotated DNA repair pathway deficiency and total number of samples/replicates with that gene KO. Next, how the total samples are divided over test and training data, and how the training data is further subdivided into 3 folds for cross-validation.

**HR**		**MMR**	**BER**	**NER**	**NHEJ**	**DS**	**DR**	**TLS**	**FA**
BARD1	BLM	EXO1	APEX1	CUL3	POLL	ATM	ALKBH2	MAD2L2	FANCA
BRCA1	BRCA2	MLH1	APEX2	CUL5	NHEJ1	ATR	ALKBH3	POLH	FANCB
BRIP1	EME1	MLH3	PARP1	ERCC1	XRCC5	ATRIP	MBD4	POLK	FANCC
GEN1	MUS81	MSH2	POLB	ERCC2	XRCC6	CHEK1	MGMT	POLN	FANCD2
NBN	PALB2	MSH3	TDG	ERCC4		RNMT		POLQ	FANCI
RAD50	RAD51	MSH6	TDP1	ERCC6		TOPBP1		REV1	FANCL
RBBP8	SHFM1	PMS1	UNG	XPA		TP53		REV3L	FANCM
TOP3A	TP53BP1	PMS2		XPC				SHPRH	UBE2T
XRCC2									

Table S1. List of repair genes included in the analysis and the corresponding pathway annotated. (HR: homologous recombination; MMR: mismatch repair; BER: base excision repair; NER: nucleotide excision repair; NHEJ:non-homologous end joining; DS: damage sensing; DR: direct repair; TLS: translesion synthesis; FA: fanconi anemia).

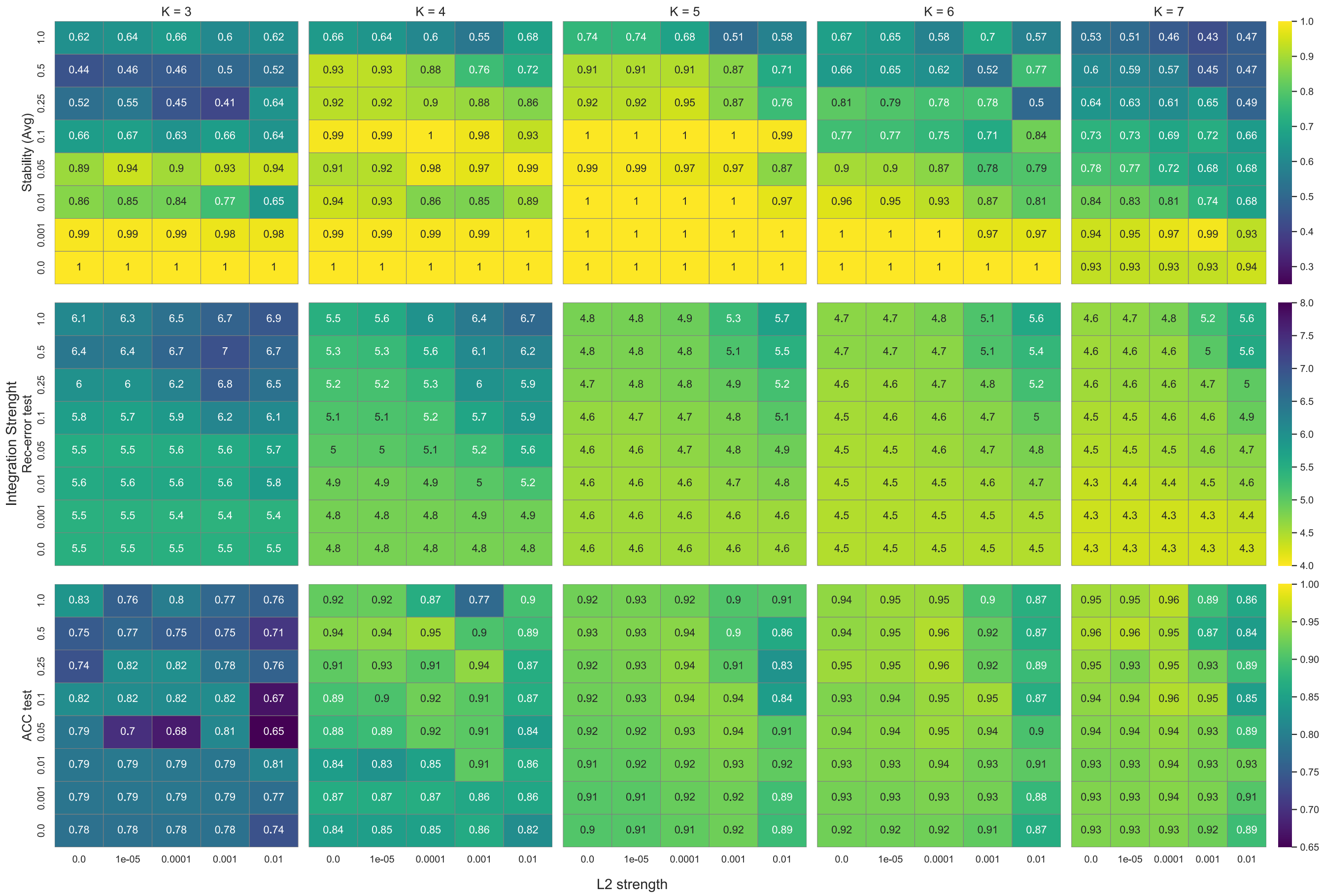

Fig. S6: **Heatmap showing hyperparameter optimization results.** First row) the average stability of the signatures. Second row) reconstruction error. Third row) prediction accuracy. Each cell in the square indicates the score for that metric of a hyperparameter setting and is annotated and colored according to the the value for the particular metric. The main columns indicate the number of signatures K, ranging from 3 to 7. Within each square, the rows indicate the integration strength, and the columns the (L2) regularization strength.

Gene:	Recall:	Y_{MMRd} (avg):	E_{MMRd} (avg):
**MLH1**	1.0 (30/30)	1.0	1.0
**MLH3**	1.0 (1/1)	1.0	1.0
**MSH3**	1.0 (3/3)	1.0	0.63
**MSH2**	1.0 (2/2)	1.0	0.67
**EXO1**	1.0 (1/1)	1.0	1.0
**Total**	**1.0** (37/37)	1.0	0.96

(A) **MMR**

Gene:	Recall:	Y_{HRd} (avg):	E_{HRd} (avg):
**BRCA2**	1.0 (6/6)	0.95	0.75
**BRCA1**	0.67 (2/3)	0.49	0.51
**RAD50**	1.0 (1/1)	1.0	0.93
**GEN1**	1.0 (1/1)	1.0	0.98
**XRCC2**	1.0 (1/1)	0.96	0.6
**Total**	**0.92** (11/12)	0.85	0.71

(B) **HR**

Table S2. Recall of repair pathway deficiency in tumours with homozygous mutated repair gene in the respective pathway.

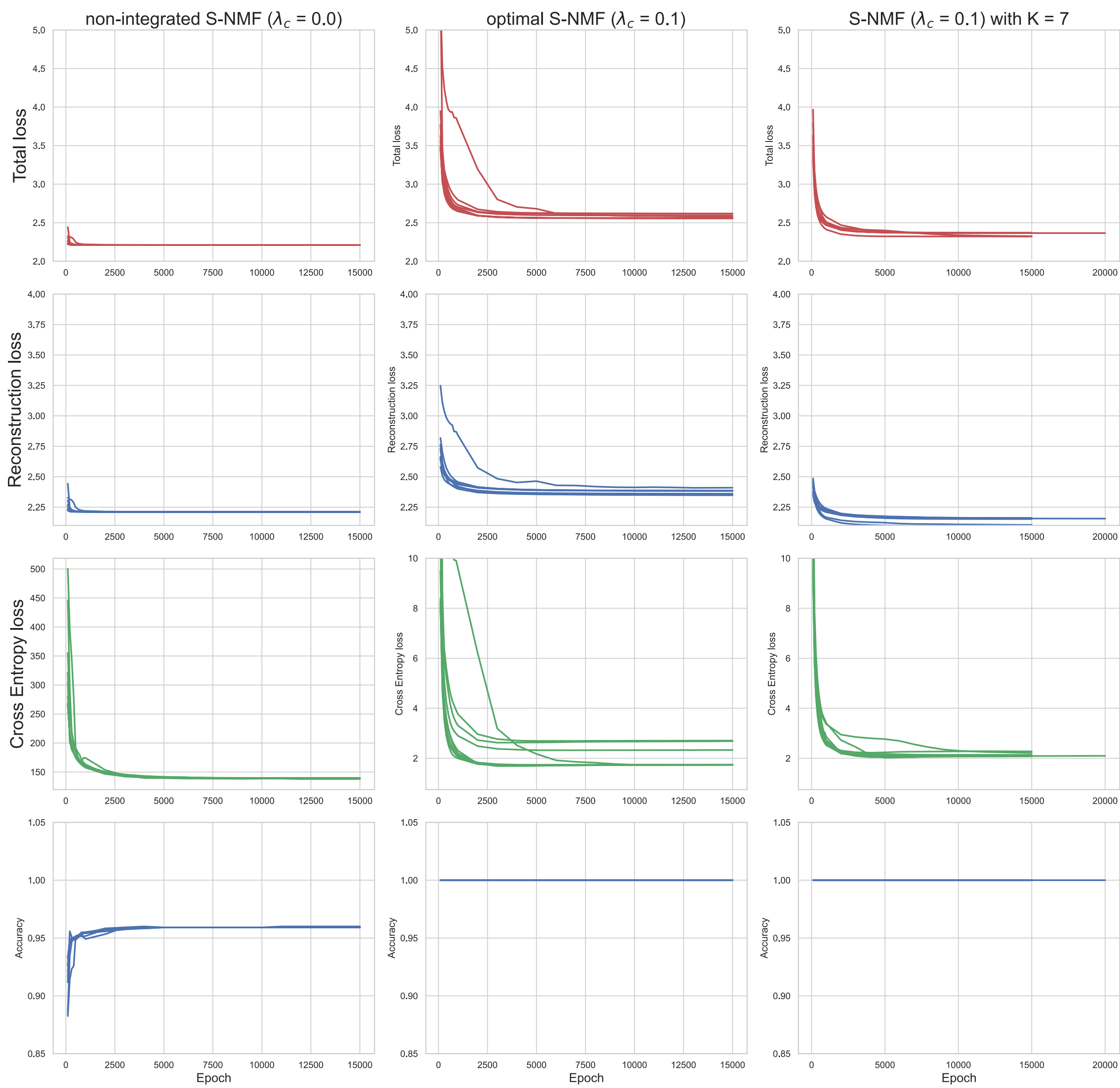

Fig. S7: **Training curves showing development of loss during the training epochs.** Left) non-integrated SNMF. Middle) optimal SNMF. Right) SNMF with K=7. Top row) total loss. 2nd row) Reconstruction loss (Frobenius reconstruction error). 3rd row) Cross-entropy loss. Bottom row) Prediction accuracy on training data.

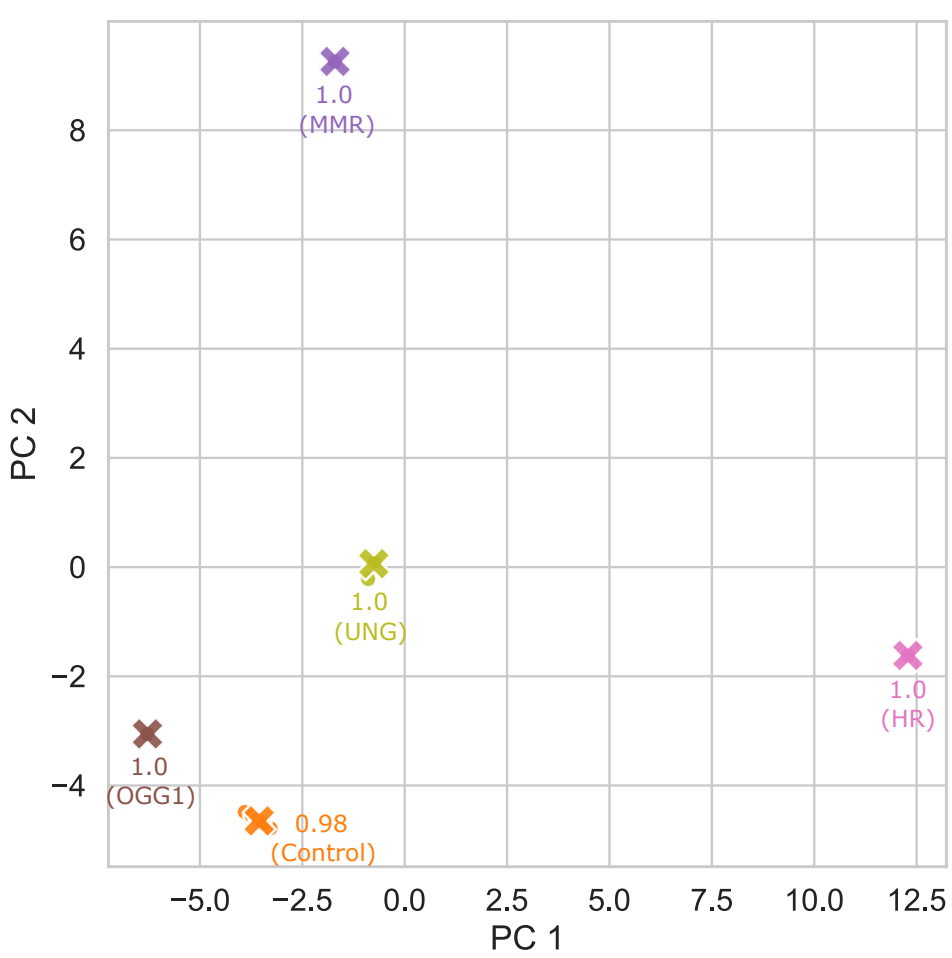

(A) Non-integrated SNMF (average stability = 1.0)

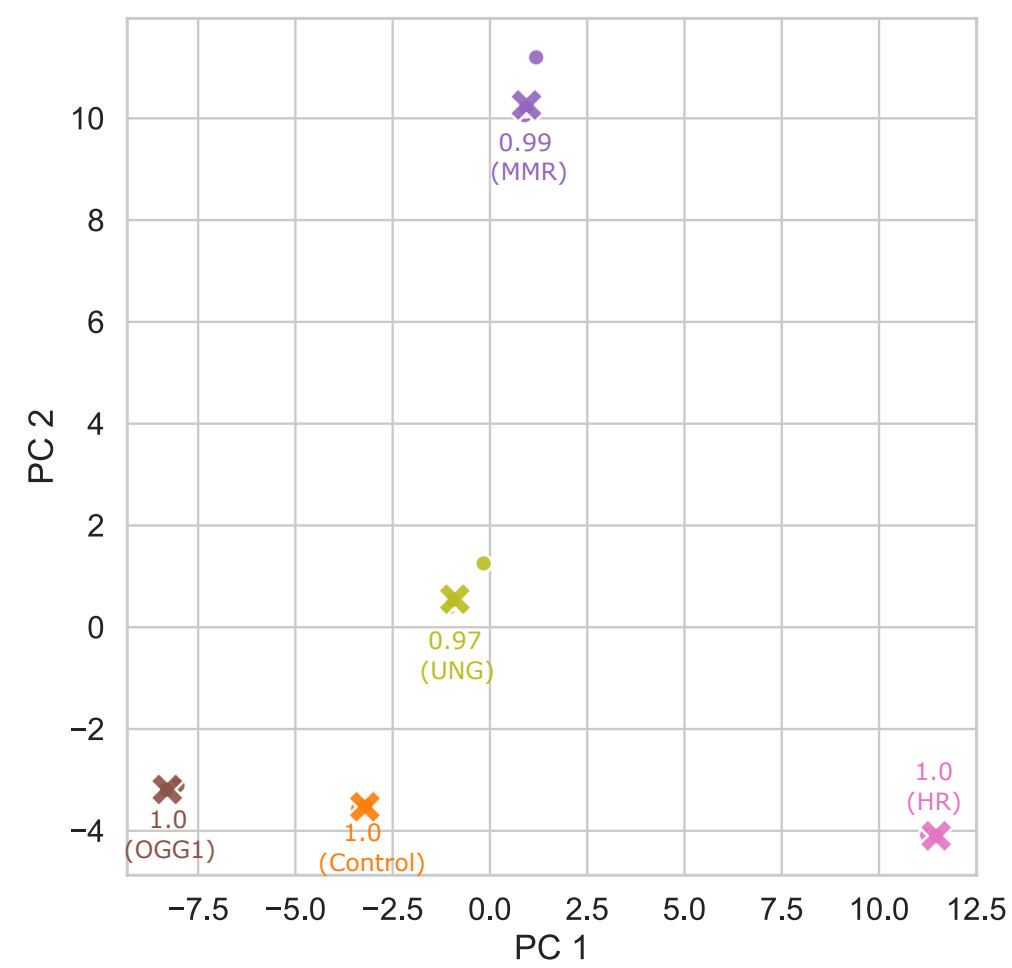

(B) integrated SNMF (average stability = 0.99)

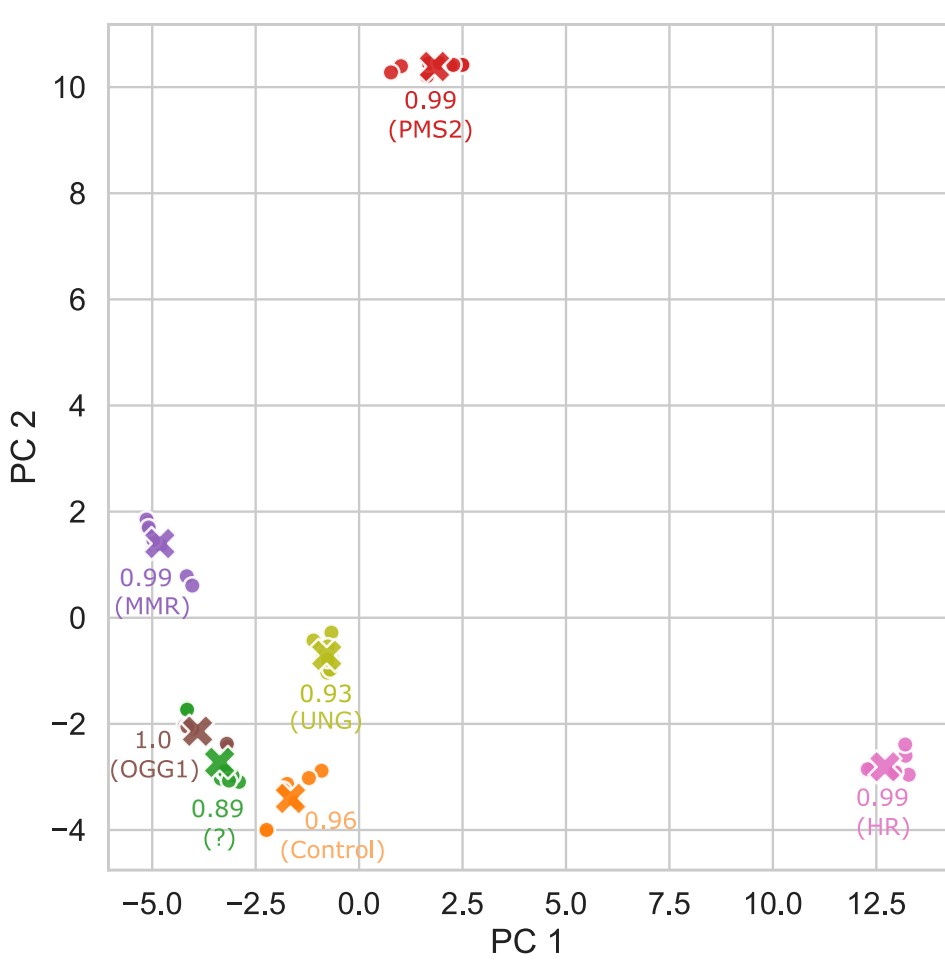

(C) Non-integrated SNMF with K = 7 (average stability = 0.95)

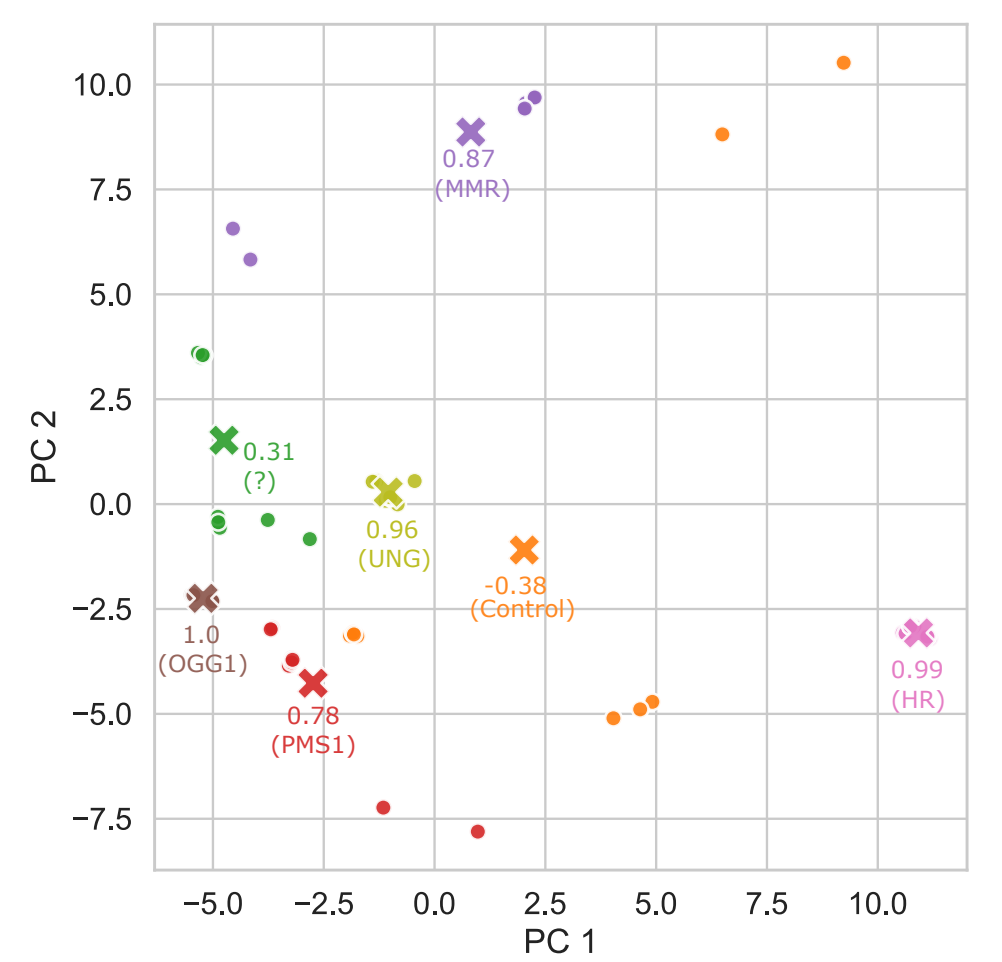

(D) integrated SNMF with K = 7 (average stability = 0.65)

Fig. S8: **PCA plot of signatures of the 10 training runs and the final signatures.** Dots (•) represent the 10 training runs, crosses (×) the centroid of each cluster (i.e. the final signatures). The colours indicate the clusters found by partition-clustering (coloring correspond to exposure in Fig. 5). Each centroid is annotated with the stability of the cluster and the repair pathway or gene KO in which the final signature had the highest exposure. Top row) $K = 5$. Bottom row) $K = 7$. Left column) non-integrated S-NMF. Right column) integrated SNMF.

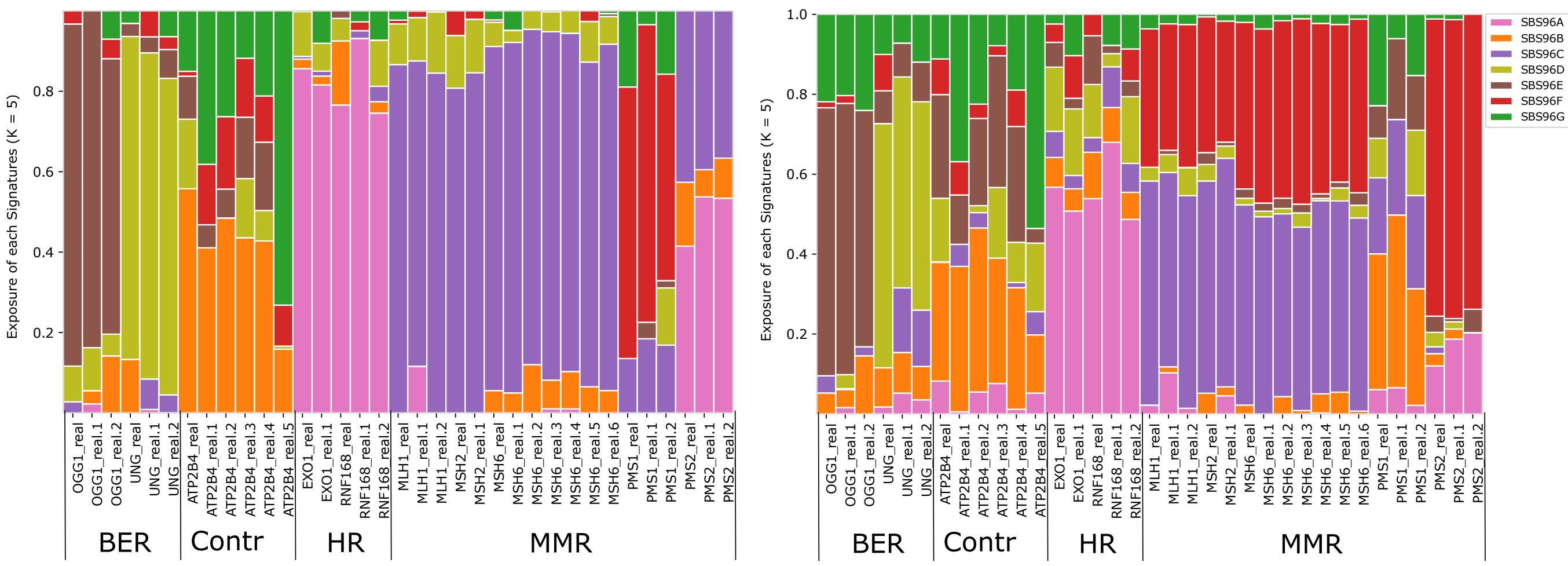

(A) Exposure found by the integrated SNMF ($\lambda_c = 0.1$) with $K = 7$. (B) Exposure found by the non-integrated SNMF ($\lambda_c = 0.0$) with $K = 7$.

Fig. S9: **Exposure of the signatures (with $K = 7$) found by SNMF**. Left) integrated SNMF. Right) non-integrated SNMF. Only the real samples are shown to allow for practical visualization.

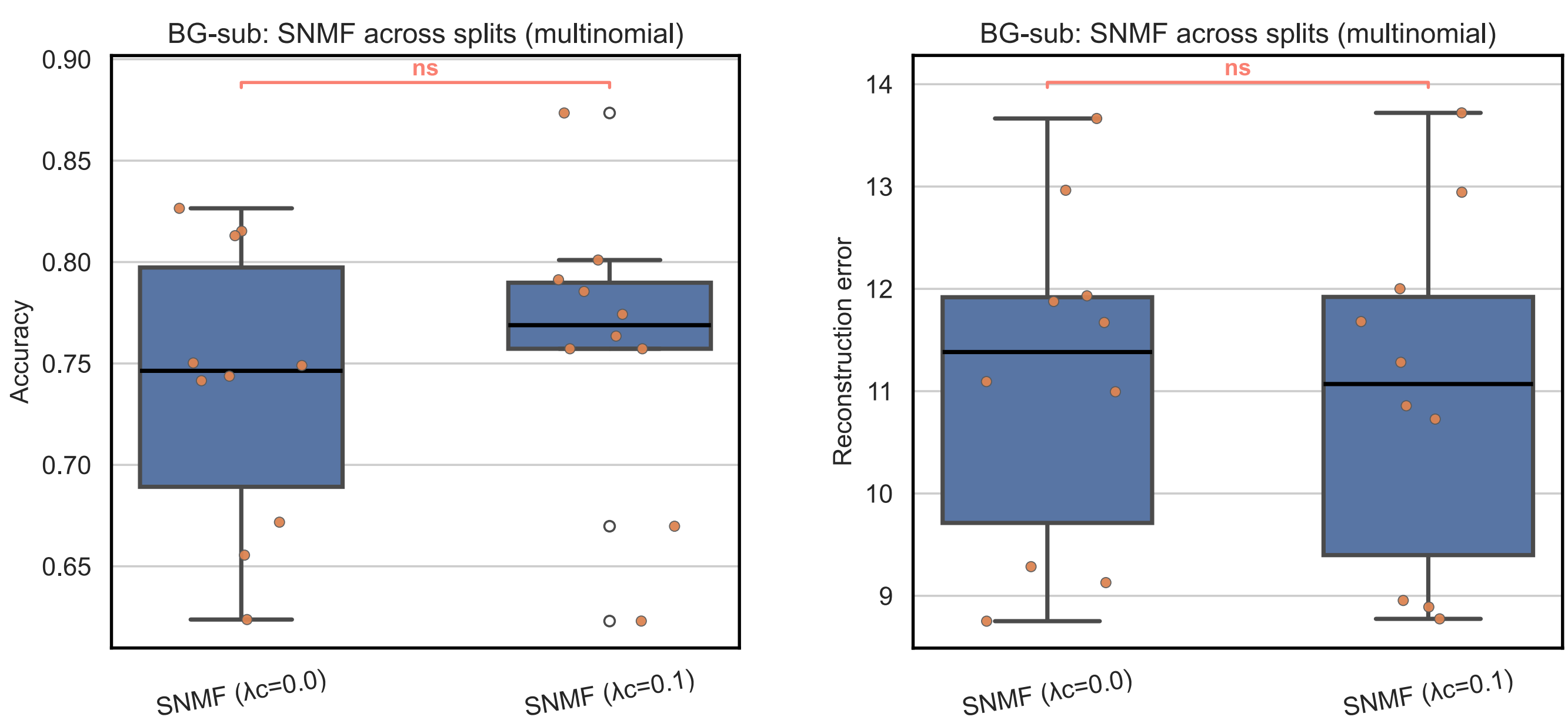

Fig. S10: **SNMF performance after subtraction of the ATP2B4d mutation profile as background. Left:** Test accuracy of non-integrated NMF ($\lambda_c = 0$) and integrated SNMF ($\lambda_c = 0.1$) after ATP2B4-background correction across data splits. **Right:** Reconstruction error for the same models and splits. Integrated SNMF retained a modest accuracy advantage over non-integrated NMF on background-subtracted data, while reconstruction performance remained comparable between settings.

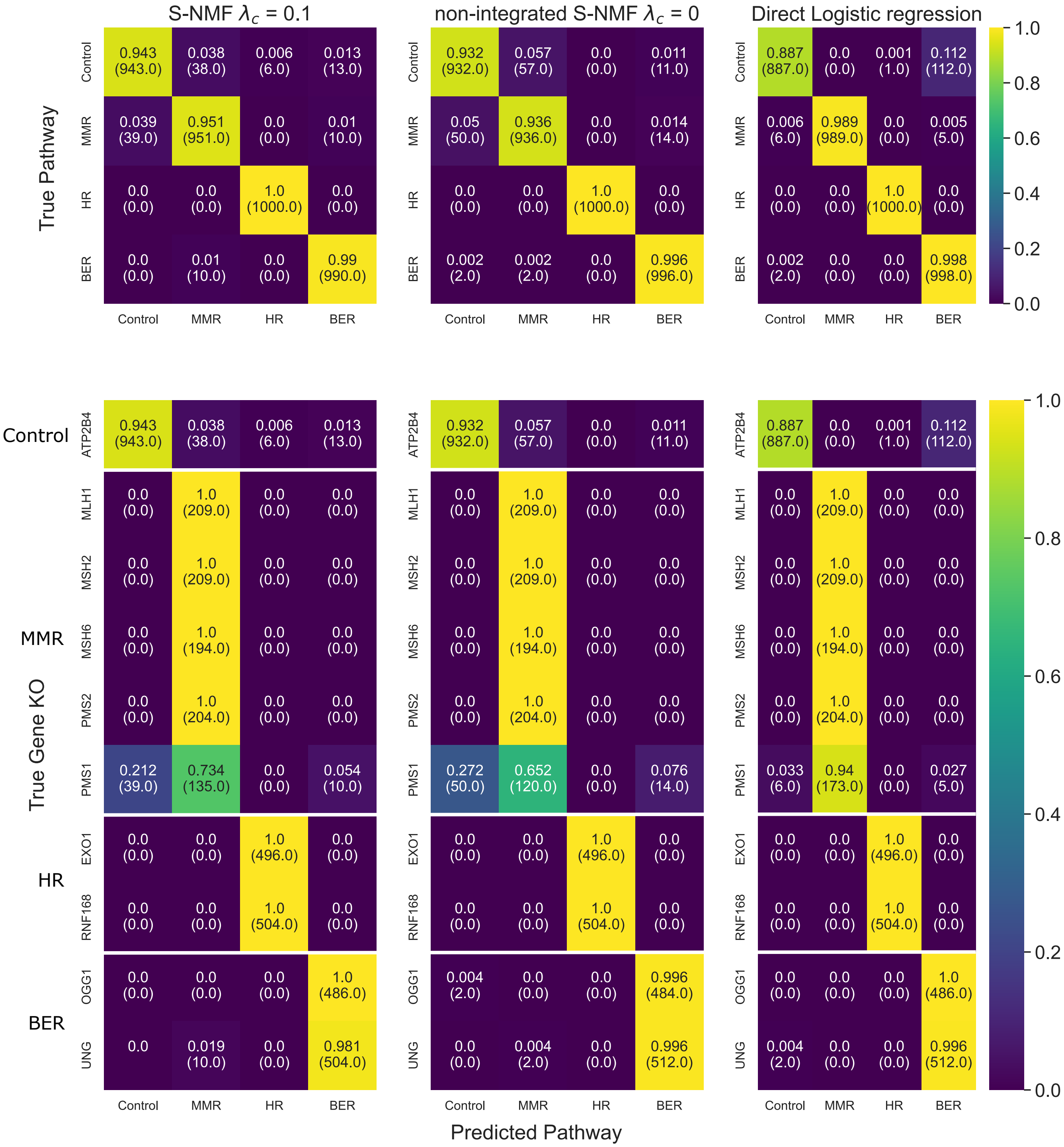

Fig. S11: **Confusion matrices with respect to pathway and gene KO.** Labeled with the percentage of samples that is predicted to the concerned pathway per true pathway/gene KO, the coloring is based on the same percentage. (In parentheses is the total number of sample with the concerned prediction) Overall prediction accuracy for integrated SNMF is 0.971, non-integrated SNMF is 0.966, and direct logistic regression 0.968.

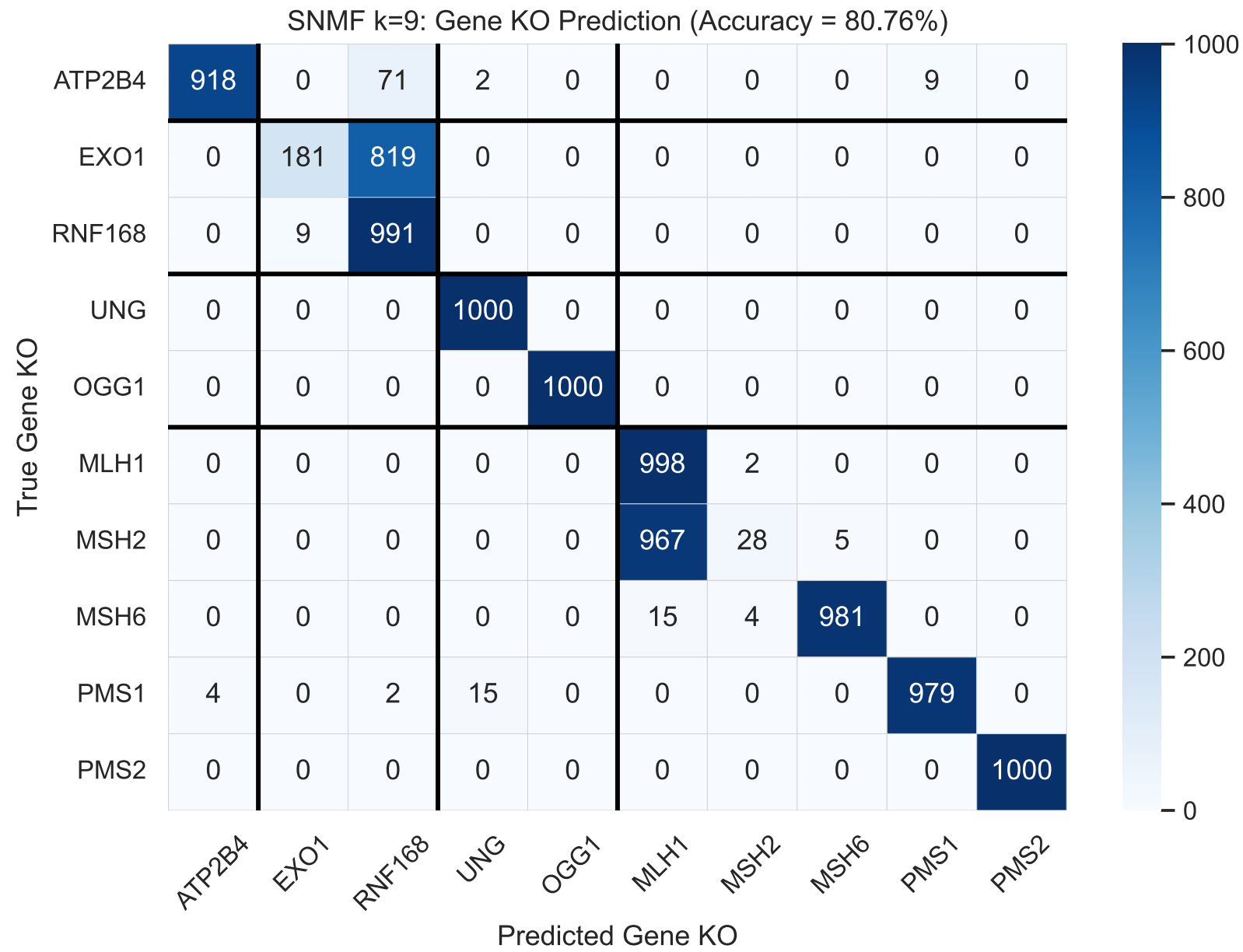

Fig. S12: **Performance of gene-level SNMF model (K = 9).**. Confusion matrix showing classification accuracy for predicted DNA repair pathways across gene knockouts.

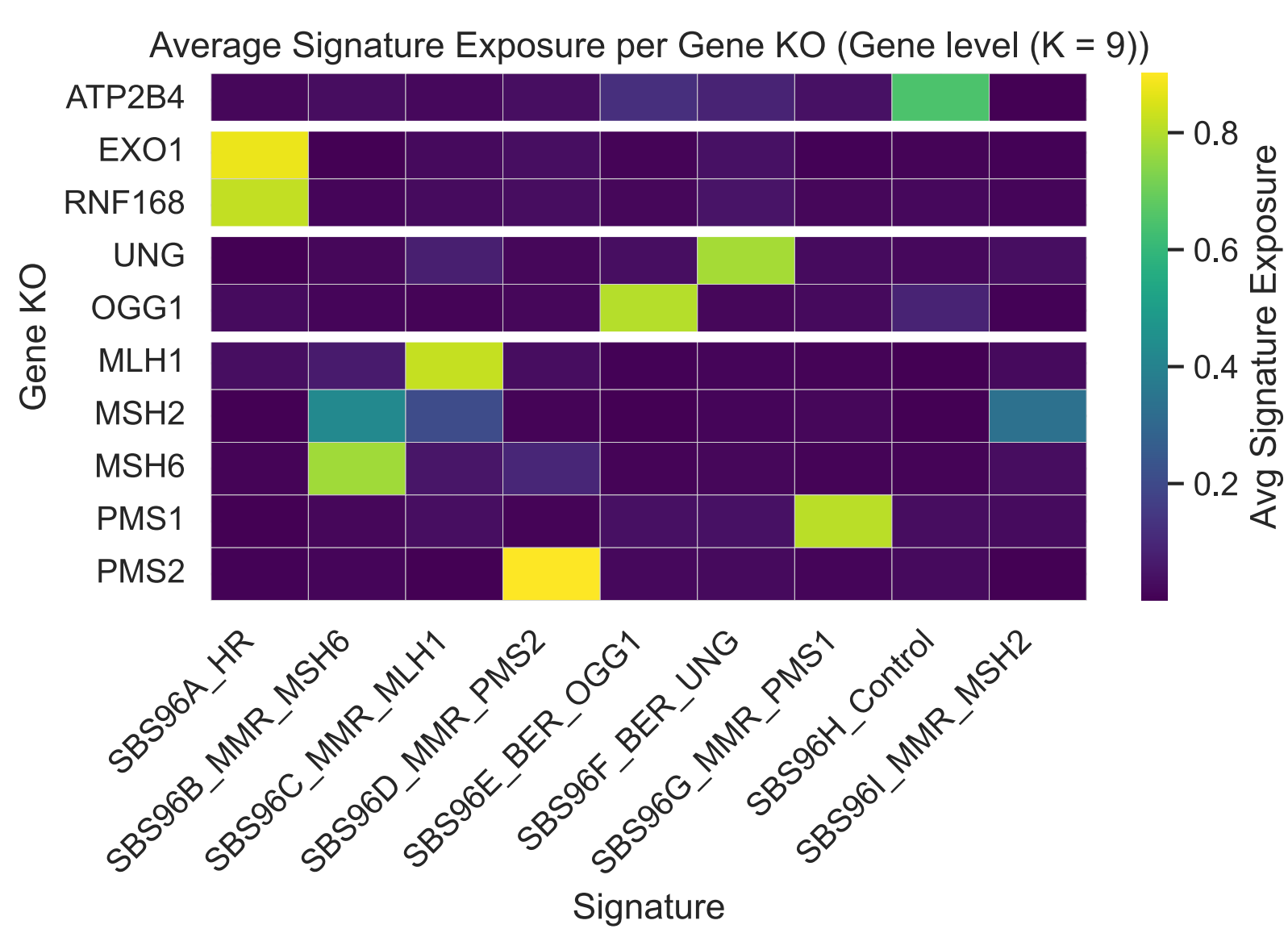

Fig. S13: **Average signature exposures across gene knockouts (gene-level SNMF, K = 9).** Each row represents a gene knockout and each column an SNMF (gene-level) signature. The signatures are annotated by their dominant (gene KO in which it has highest exposure) DNA repair pathway and gene.

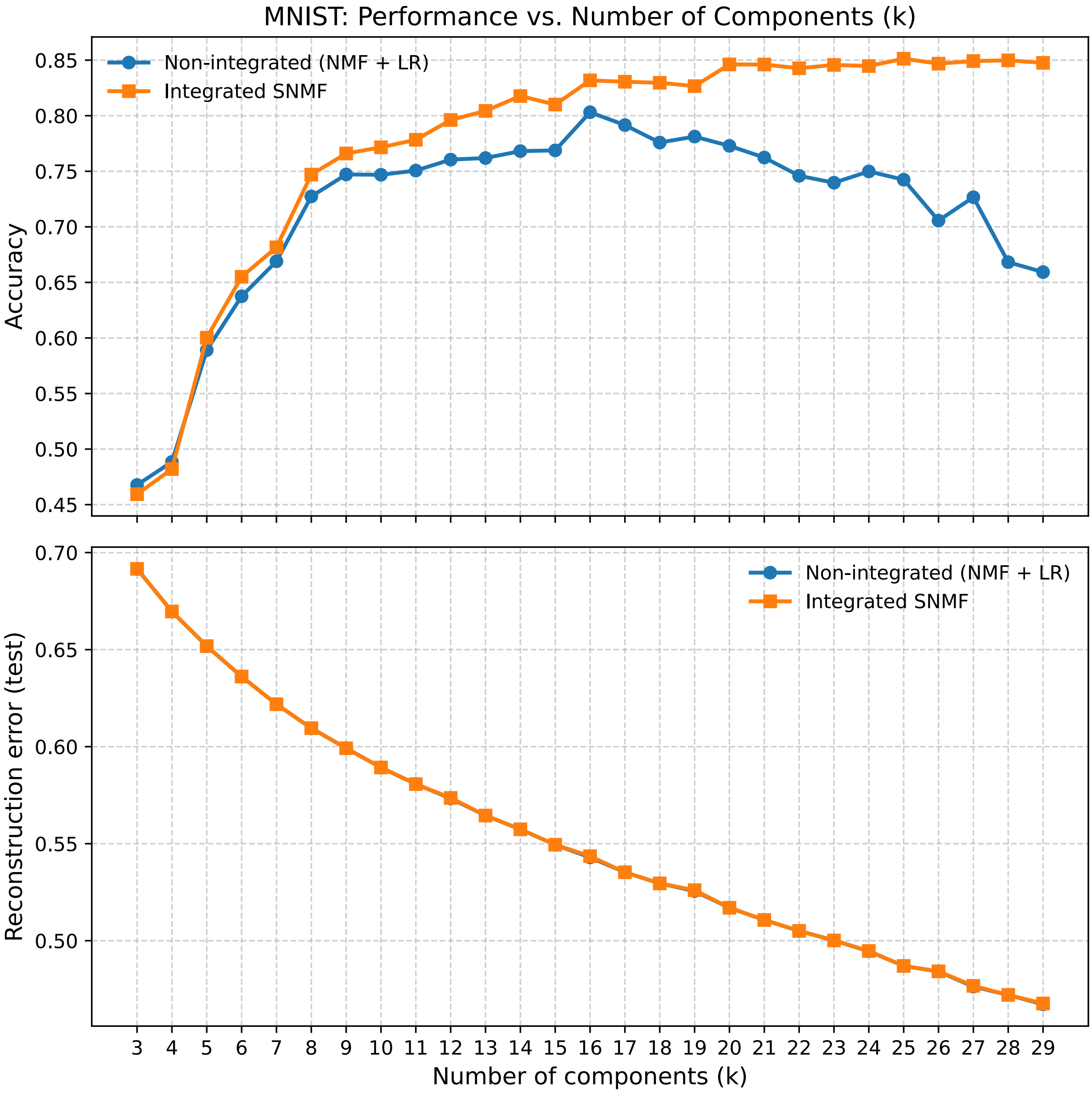

Fig. S14: **Performance of SNMF and conventional NMF on the MNIST hand-written digit dataset. Top)** Prediction accuracy. **Bottom)** Reconstruction error.

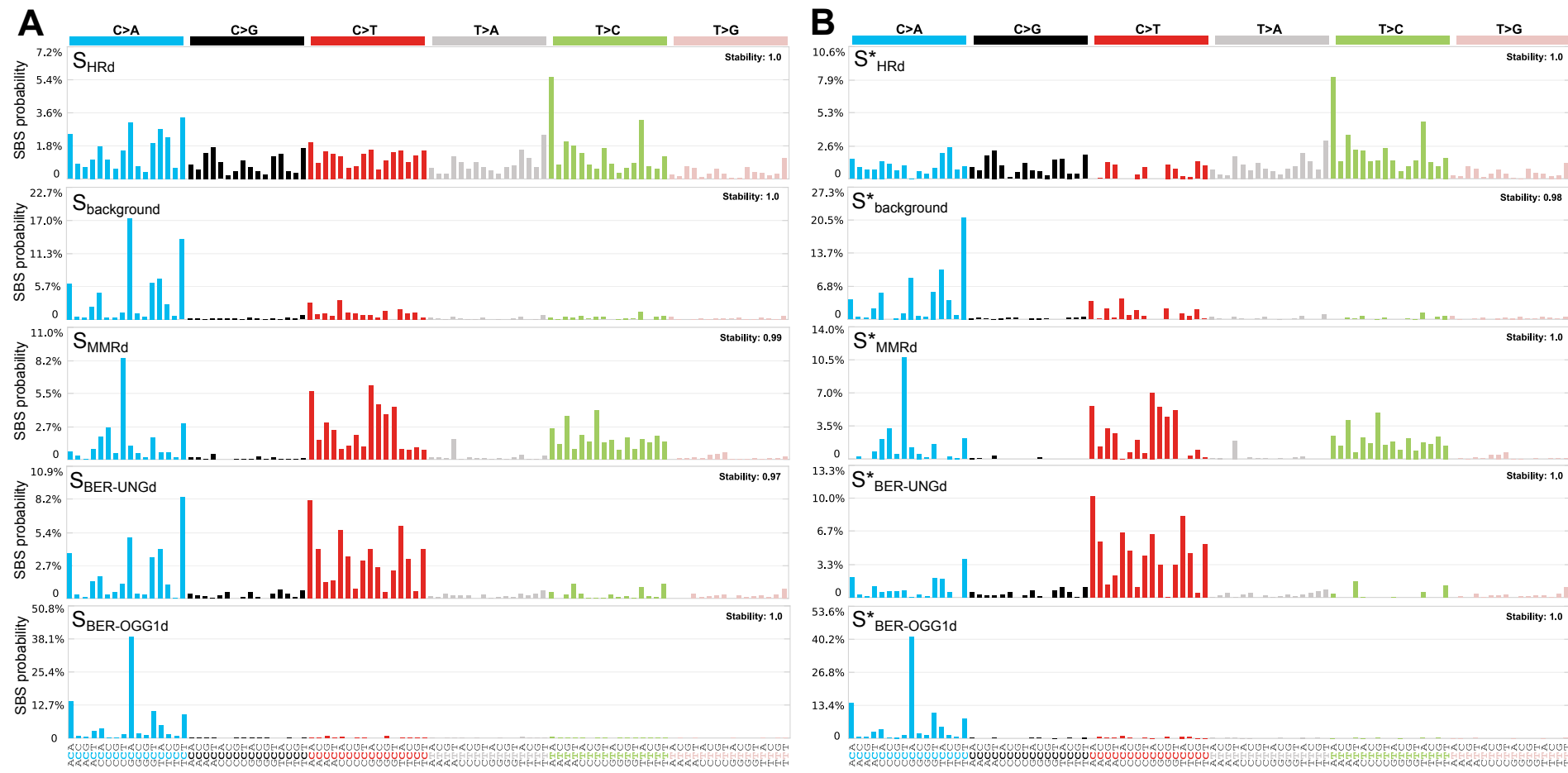

Fig. S15: **Cell line signatures identified by integrated SNMF and NMF (A-B)** Signatures found by (A) integrated SNMF and unsupervised NMF (B) in the cell line samples.

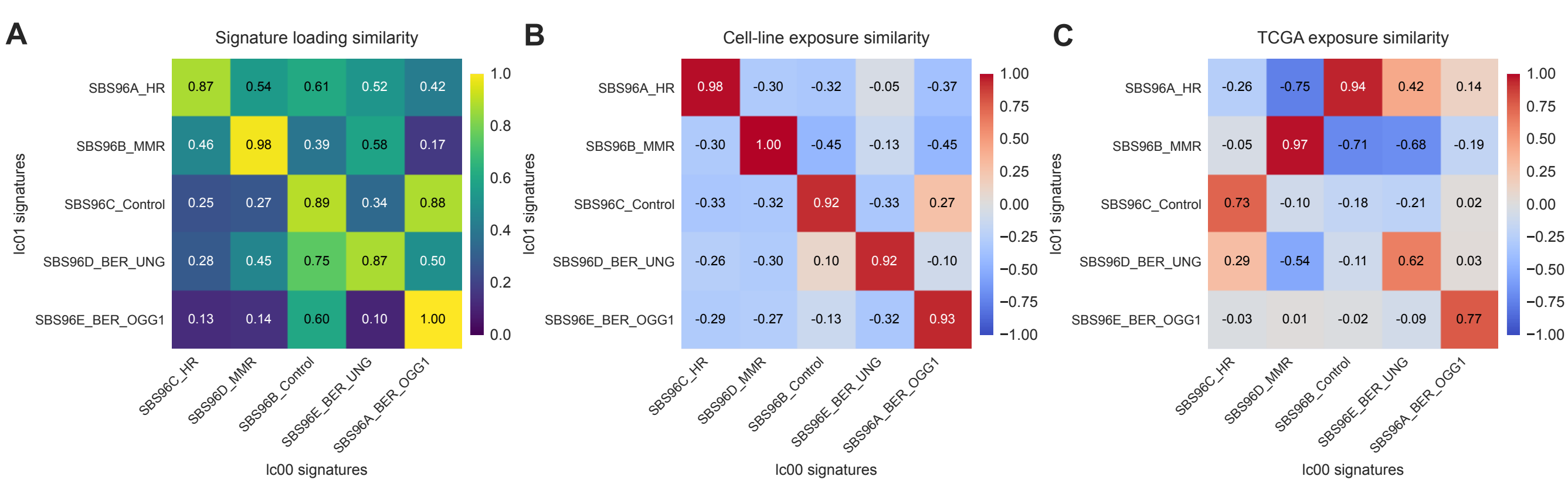

Fig. S16: **Comparison of signatures and exposures learned with unsupervised NMF and SNMF.** Similarity between the five signatures learned without label coupling ($\lambda_c = 0.0$; lc00, columns) and with label coupling ($\lambda_c = 0.1$; lc01, rows). Signatures were matched and ordered using the Hungarian algorithm to maximize cosine similarity. **A)** Cosine similarity between mutational signature profiles. **B)** Pearson correlation between signature exposures across cell-line samples. **C)** Pearson correlation between signature exposures after transfer to TCGA tumour samples. Higher diagonal values indicate that the corresponding lc00 and lc01 signatures have similar mutational profiles or are assigned to similar samples.

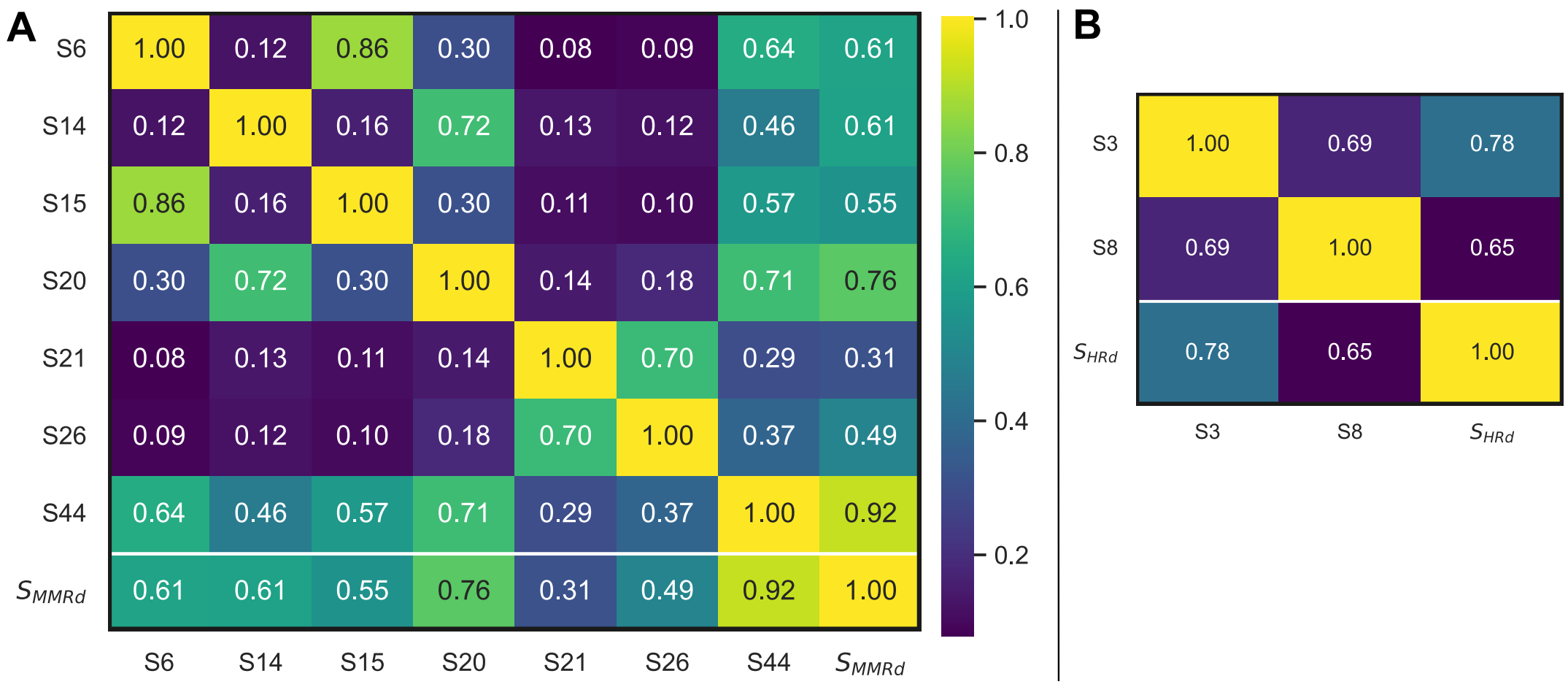

Fig. S17: **Cosine similarity between MMRd-related COSMIC signatures (S) and the SNMF signature related to the same repair pathway deficiency. A)** MMRd-related COSMIC signatures and SNMF MMRd signature (S_{MMRd}). **B)** HRd related COSMIC signatures and SNMF HRd signature (S_{HRd}).

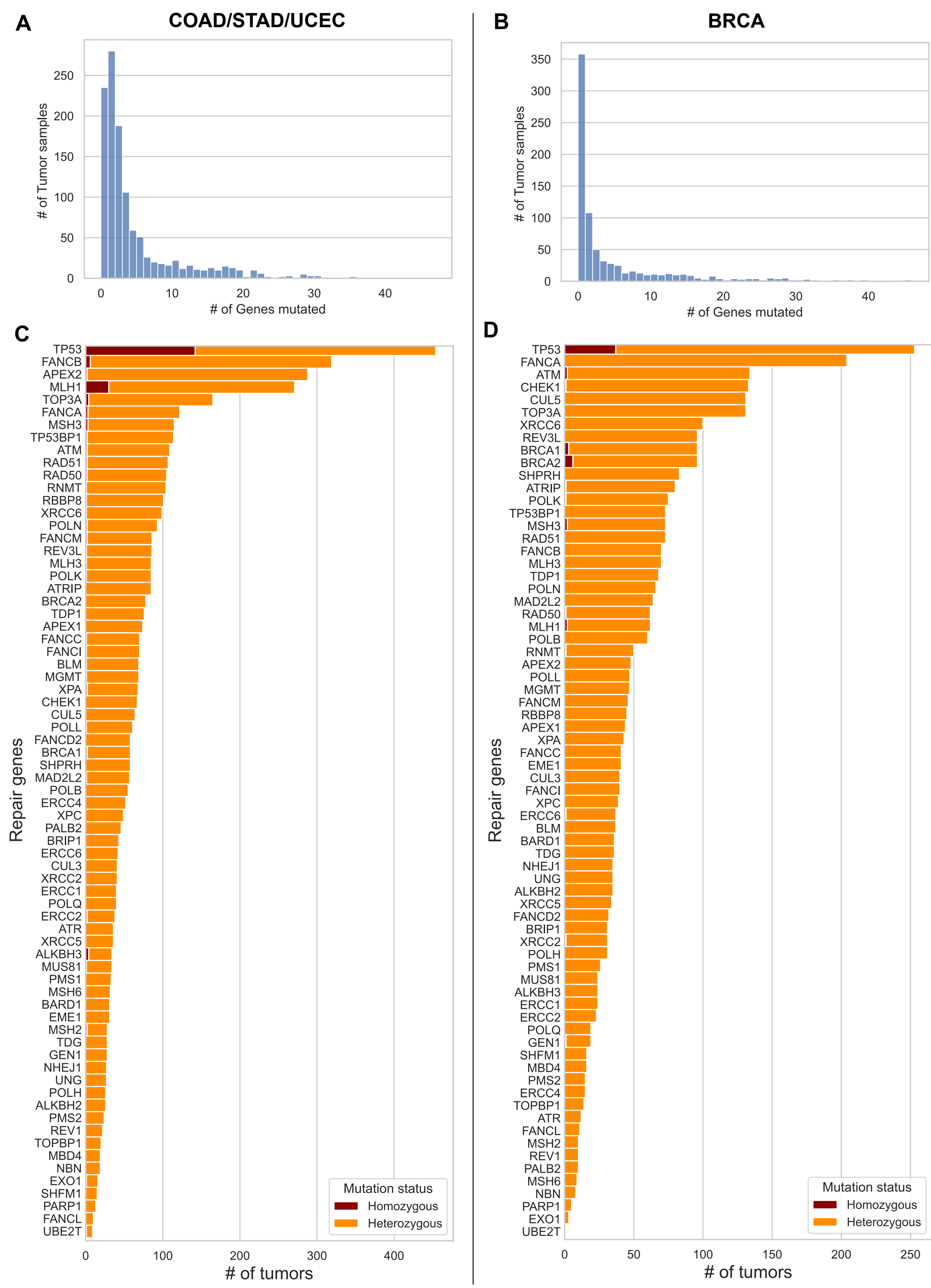

Fig. S18: **Analysis of number of mutated genes. Left)** MMRd related cancer types (in total 1176 tumour samples). **Right)** HRd related cancer types (in total 791 tumour samples). **A and C)** Histogram showing the number of mutated genes. **B and D)** For each gene, the number of tumours that had a (heterozygous or homozygous) loss of function mutation.

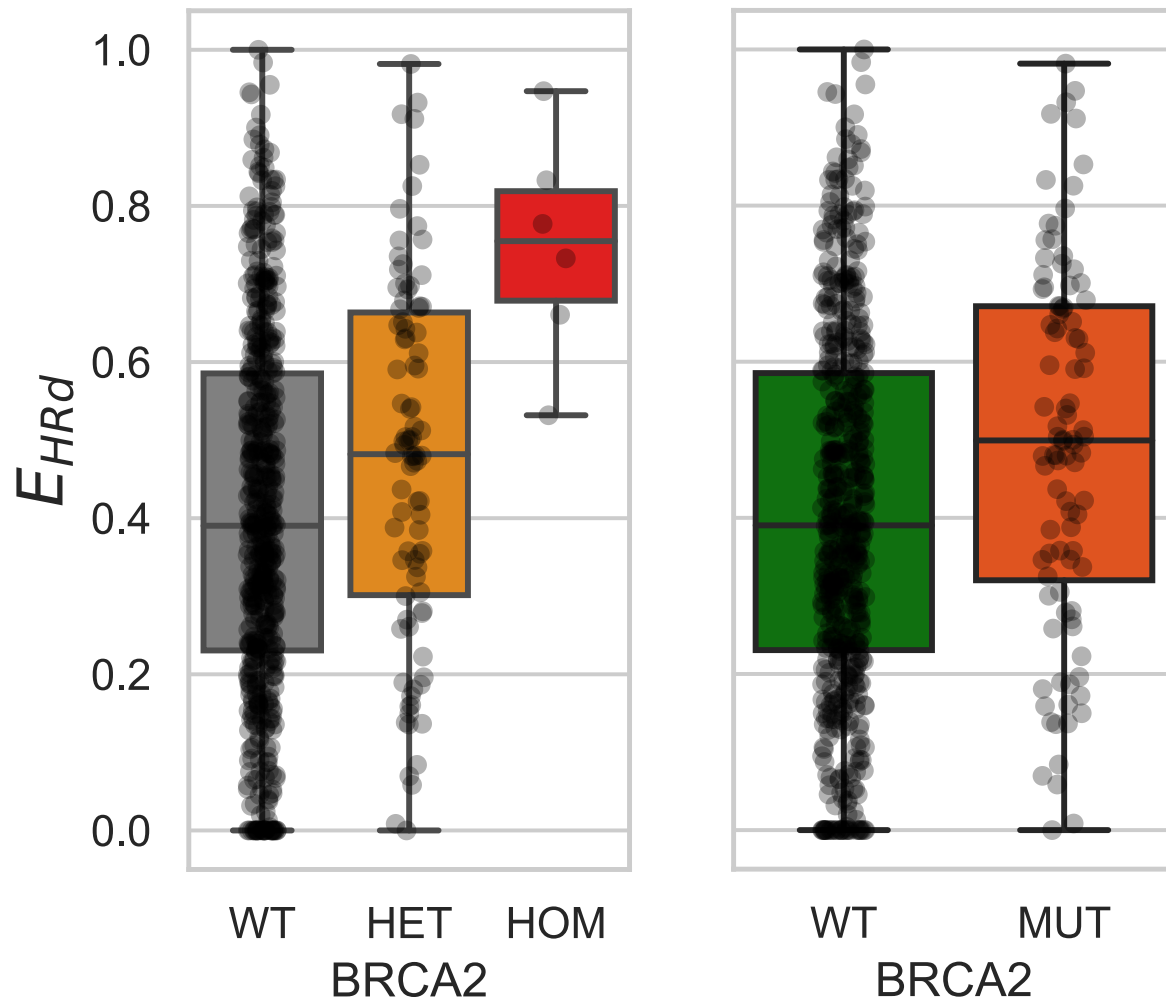

Fig. S19: **Boxplot showing the exposure of the HRd signature for BRCA2 tumours. Left)** All tumours split into three groups based on BRCA2, WT (grey) vs heterozygous (orange) vs homozygous (red) mutated. **Right)** the two groups used in the MW U test where WT has no homozygous mutation in any HR repair gene (green) and combining HET + HOM into single group of mutated tumours (dark orange).

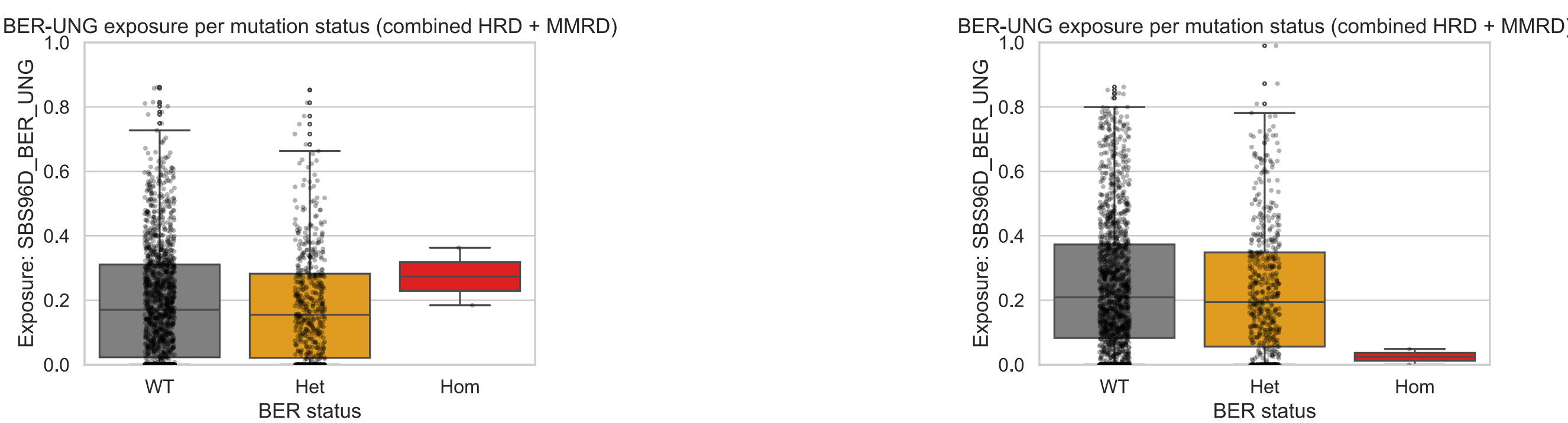

Fig. S20: **tumour exposures to BER/UNG-associated signatures.** Boxplots showing tumour-level exposures to the **(left)** SNMF-learned and **(right)** NMF-learned BER/UNG-associated (UNG^{d}) cell line signatures, colored by BER gene mutation status.

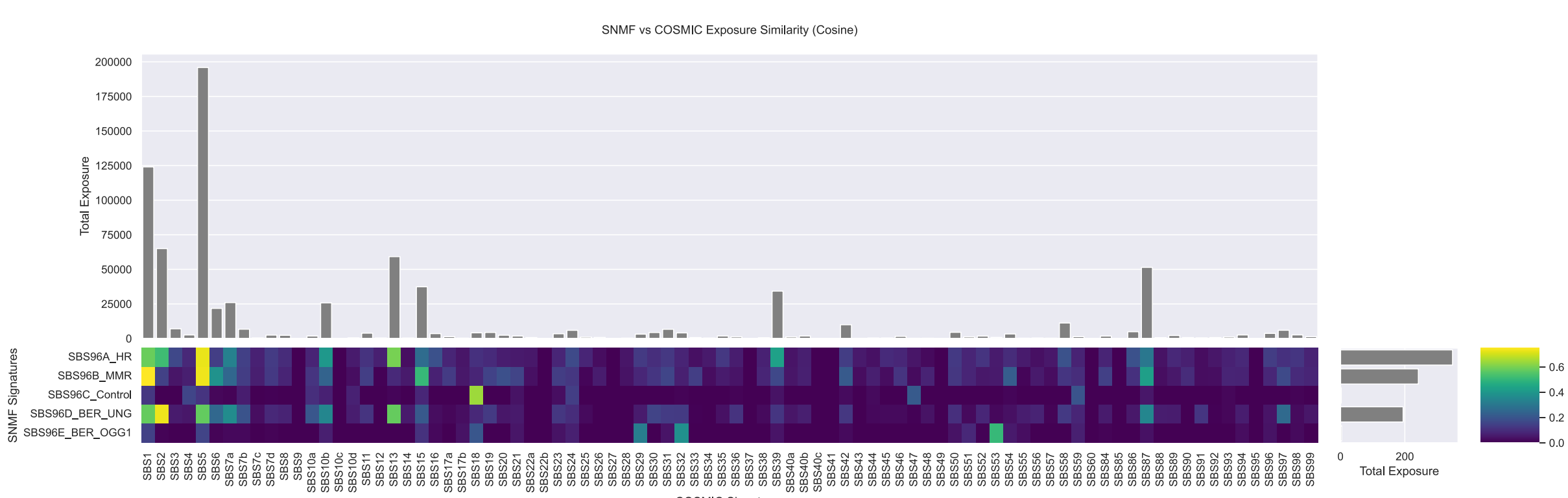

Fig. S21: **Cosine similarity between SNMF and COSMIC exposures in TCGA tumours.** Each cell represents the cosine similarity between SNMF-inferred and COSMIC reference exposures across tumour samples. Total exposure per signature is shown on the margins, and red boxes highlight the most similar COSMIC signature for each SNMF exposure profile.

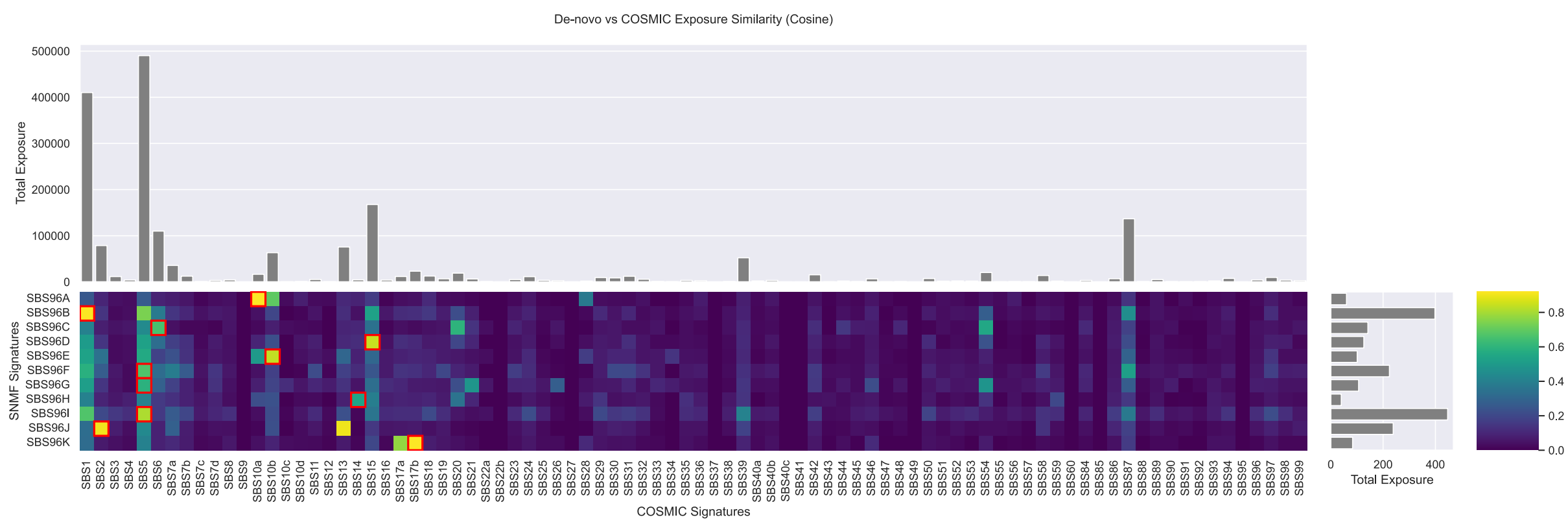

Fig. S22: **Cosine similarity between de novo NMF and COSMIC signatures in TCGA tumours.** Heatmap showing pairwise cosine similarity between de novo extracted and COSMIC reference signatures. Margins display total exposure per signature, with red boxes marking the most similar COSMIC counterpart to each de novo NMF profile.

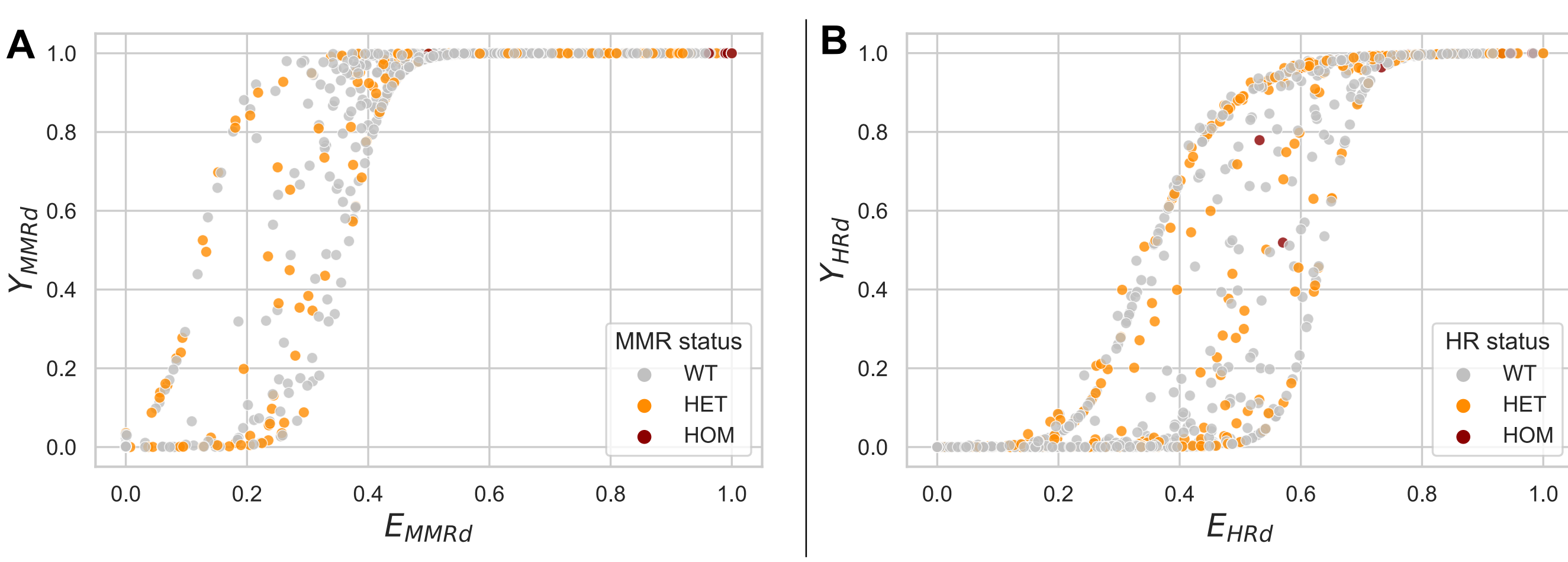

Fig. S23: **Comparing exposure (x-axis) to prediction probability (y-axis) of repair pathway deficiency for all tumours.** Indication of the decision boundary of the SNMF model on the exposure of **A)** MMR-d signature in MMR-related cancer types (COAD/STAD/UCEC) **B)** HR-d signature in HR-related cancer type (BRCA).

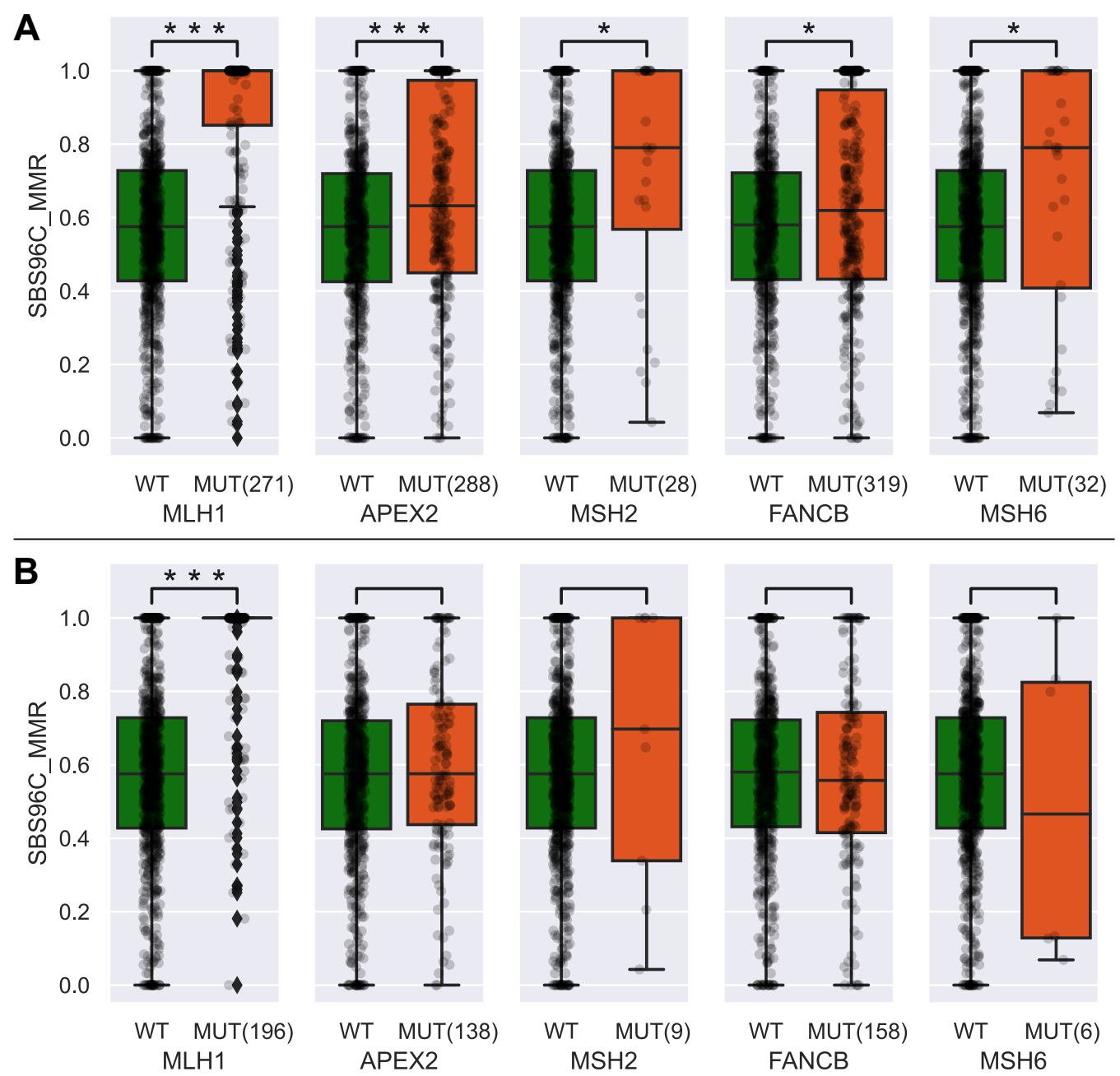

Fig. S24: **Boxplot showing effect of gene on MMRd signature exposure for genes that had a significant association.** Wild-type (WT, green) and mutated samples (MUT, orange, with number of samples): *** $p \leq 0.001$; ** $p \leq 0.01$; * $p \leq 0.05$. **Top)** Original comparison. **Bottom)** Only considering tumours without additional MMR gene mutated.

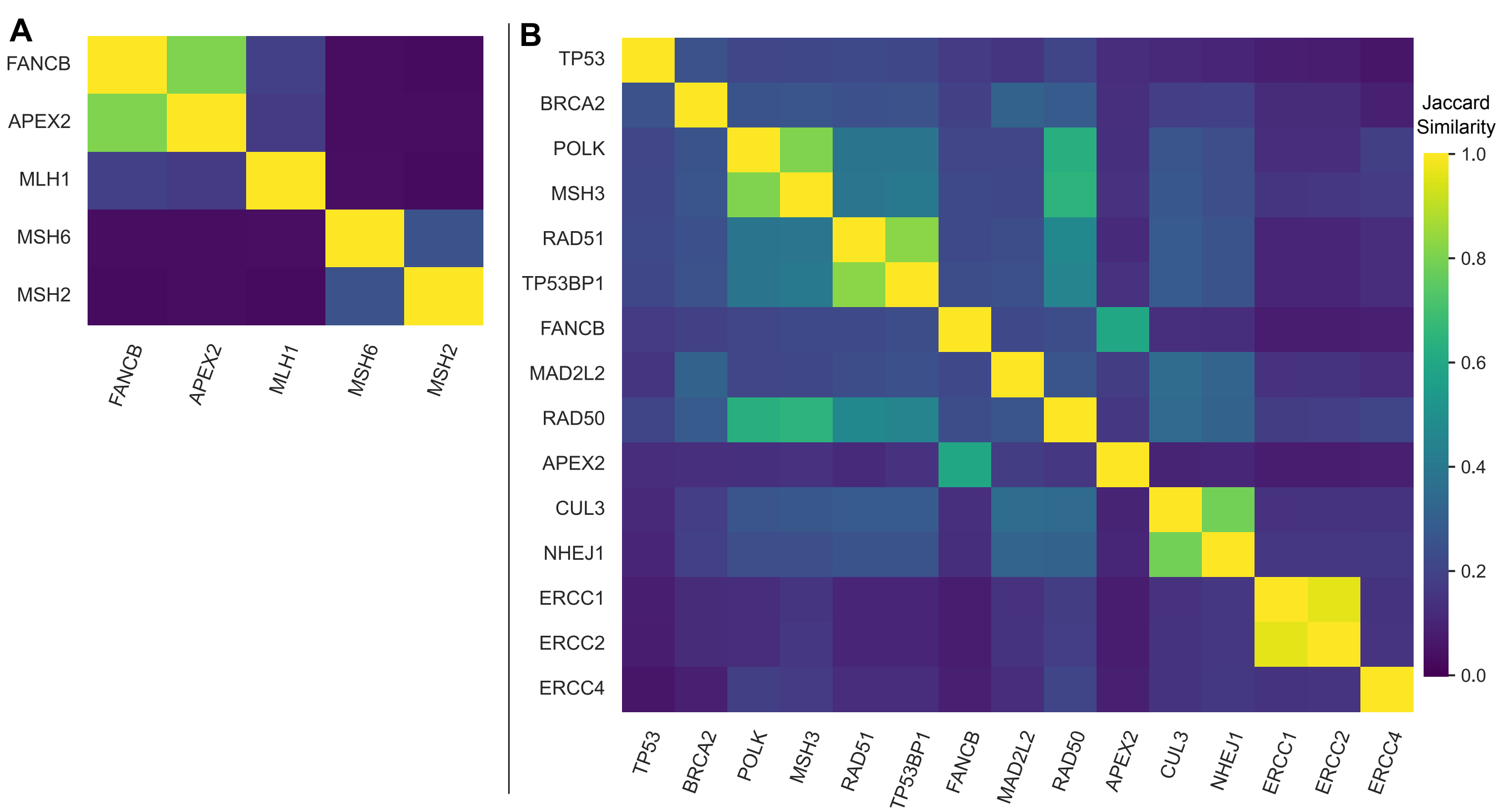

Fig. S25: **Co-occurence (measured by Jaccard Similarity (JS)) between the genes that had a significant effect on the respective pathway deficiency.**. **A)** Co-occurrence (JS) of MMRd associated genes in COAD/STAD/UCEC tumour samples. **B)** Co-occurrence (JS) of HRd associated genes in BRCA tumour samples.

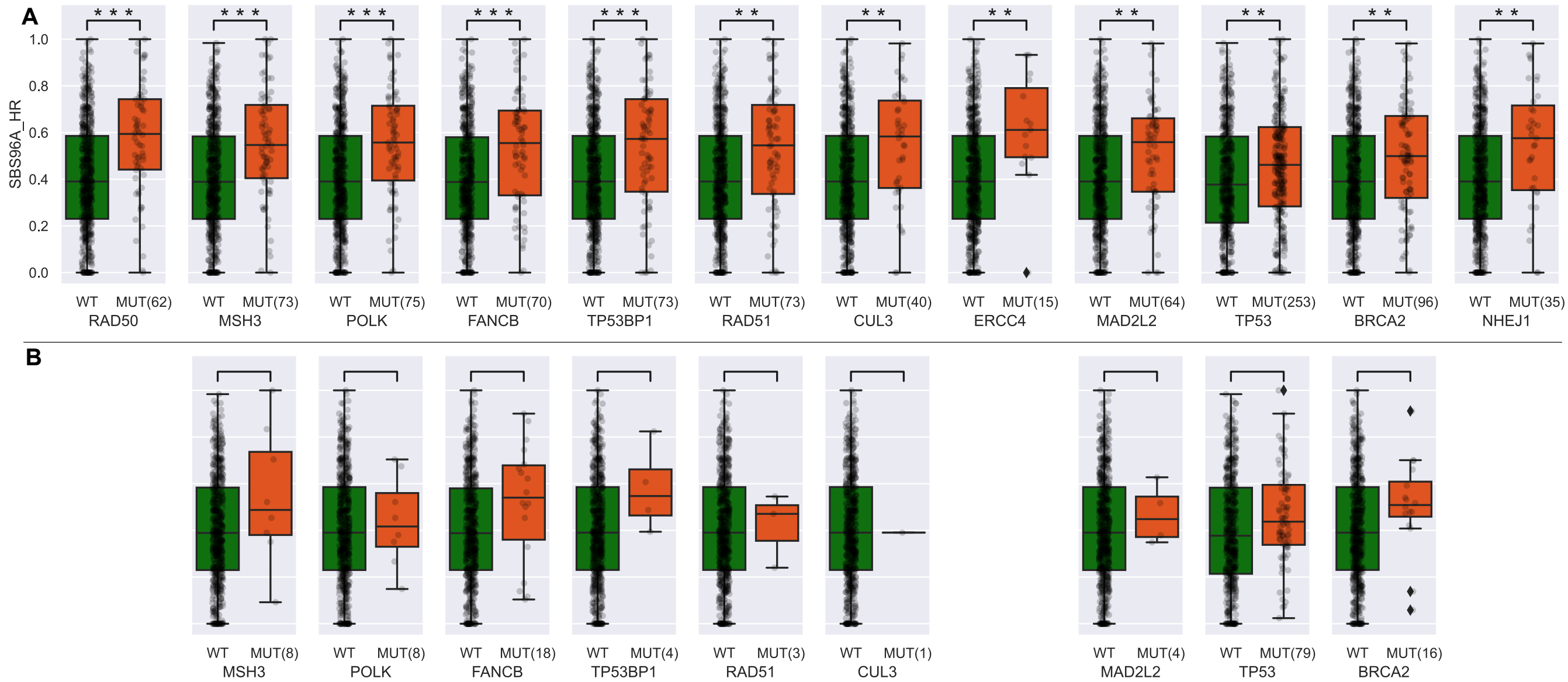

Fig. S26: **Boxplot showing effect of gene on HRd signature exposure for genes that had a significant association.** Wild-type (WT, green) and mutated samples (MUT, orange, with number of samples): *** $p \leq 0.001$; ** $p \leq 0.01$. **Top)** Original comparison. **Bottom)** Only considering tumours without additional HR gene mutated. Empty spots mean there were no tumour samples that did not have an additional mutated HR gene for that gene.

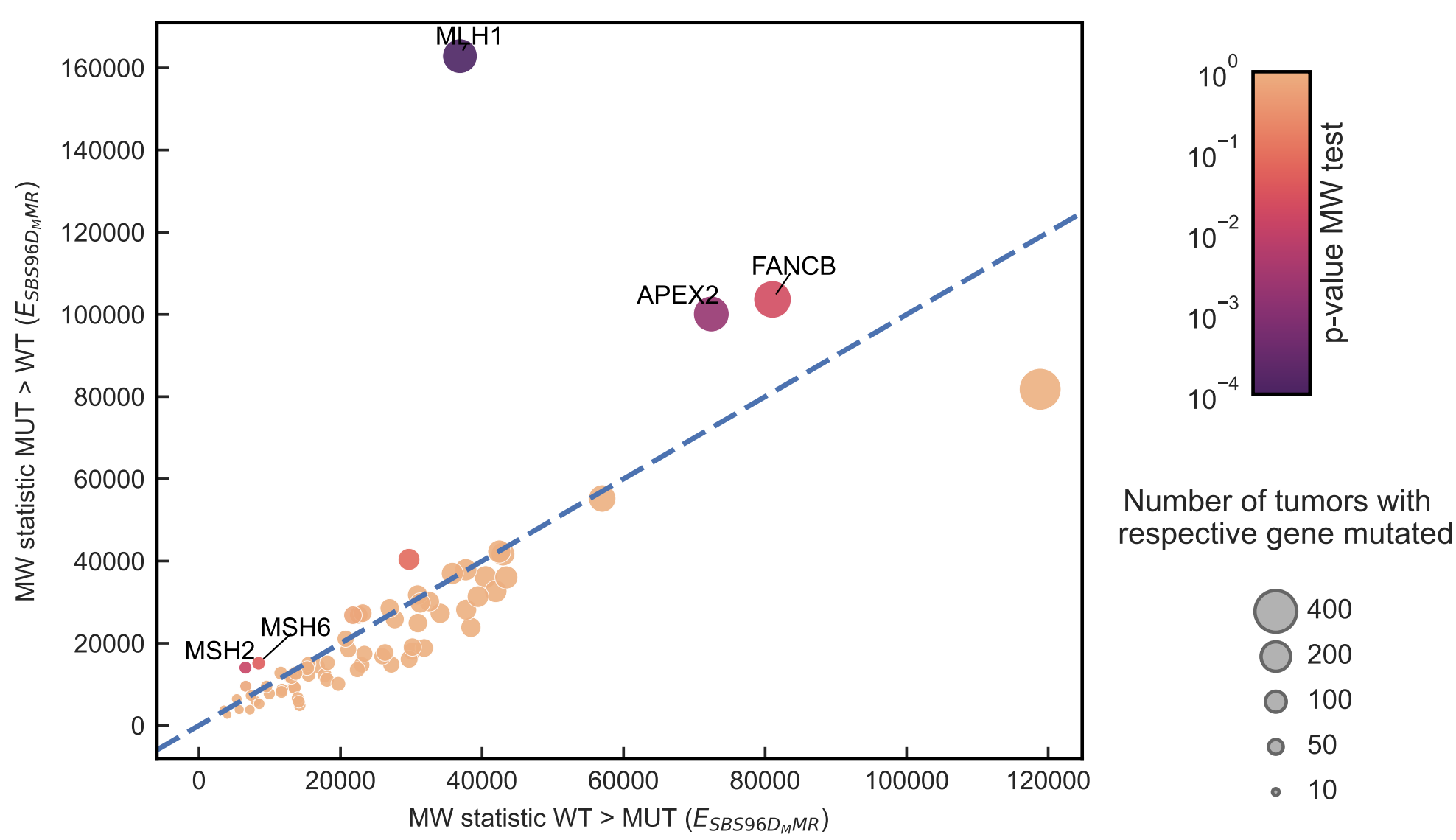

Fig. S27: **Matched non-integrated NMF transfer analysis for MMRd tumours.** Mann–Whitney U test statistics for the non-integrated NMF MMRd-associated signature fitted to COAD, STAD, and UCEC tumours. Labeled genes pass FDR < 0.05. The same significant MMR-associated genes were recovered as in the SNMF analysis, indicating that the strong MMRd signal is captured similarly by the matched unsupervised baseline.

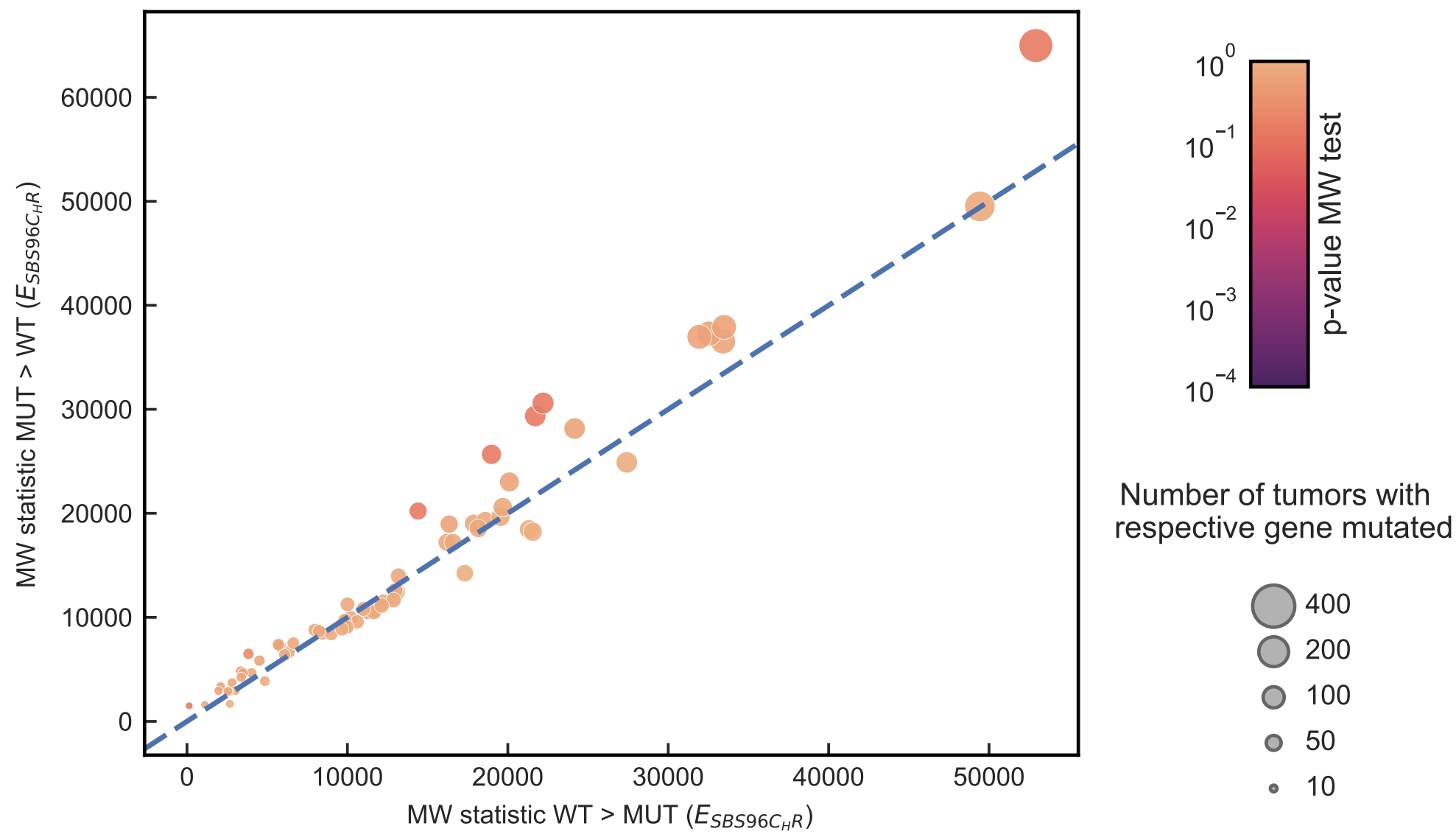

Fig. S28: **Matched non-integrated NMF transfer analysis for the HRd-associated signature in BRCA tumours.** Mann–Whitney U test statistics for the non-integrated NMF HRd-associated signature fitted to BRCA tumours. Labeled genes pass FDR < 0.01. Unlike the SNMF HRd signature, this NMF HRd-associated signature did not recover the strongest HR-related tumour gene associations.

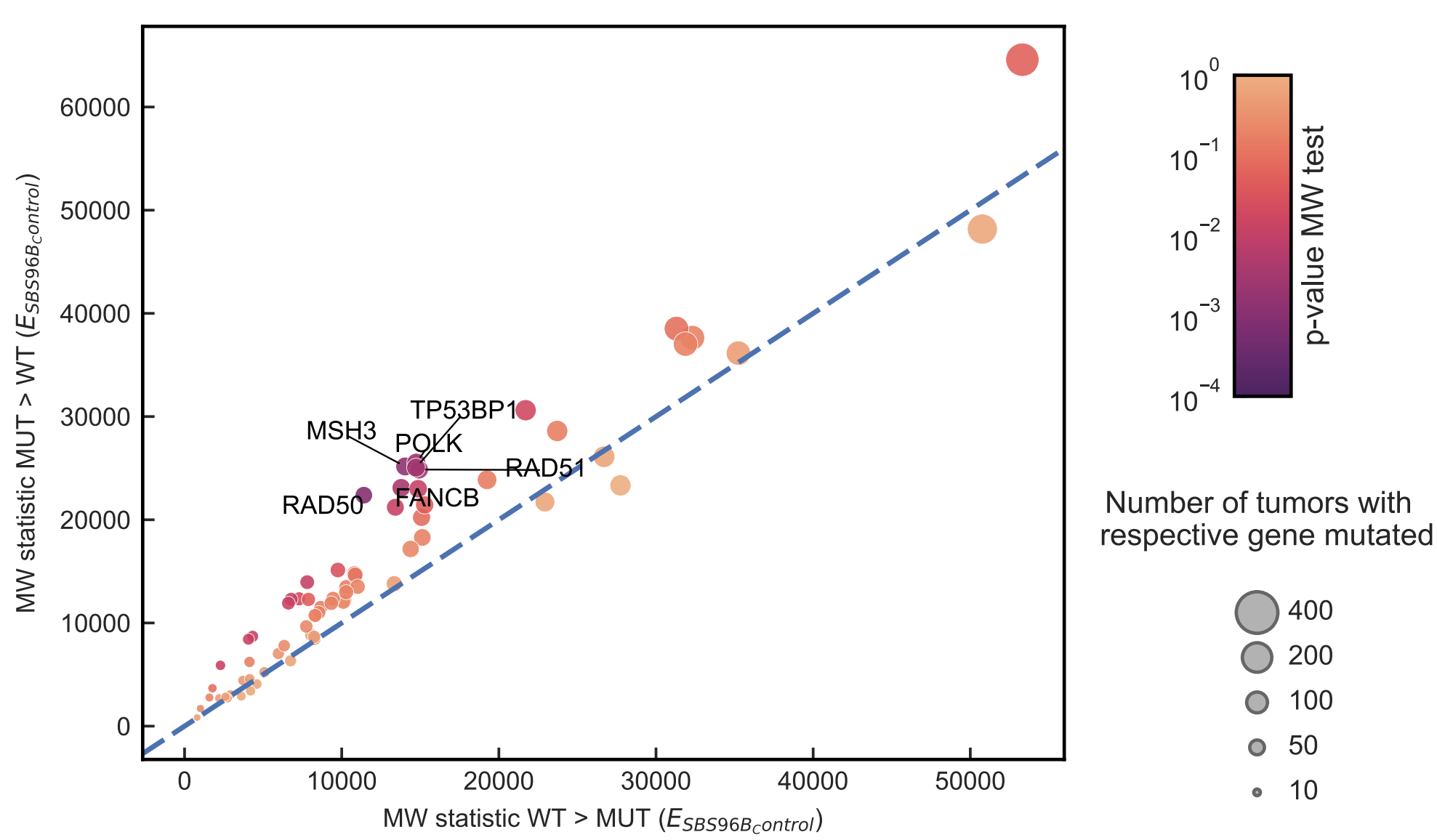

Fig. S29: **Matched non-integrated NMF transfer analysis for the control/background-associated signature in BRCA tumours.** Mann–Whitney U test statistics for the non-integrated NMF control/background signature fitted to BRCA tumours. Labeled genes pass FDR < 0.01, however no genes were significant below this threshold. These genes overlap with the HR-associated genes identified for the SNMF HRd signature in the main analysis, suggesting that the unsupervised NMF method captures related tumour variation but assigns it to a background/control-labelled signature.

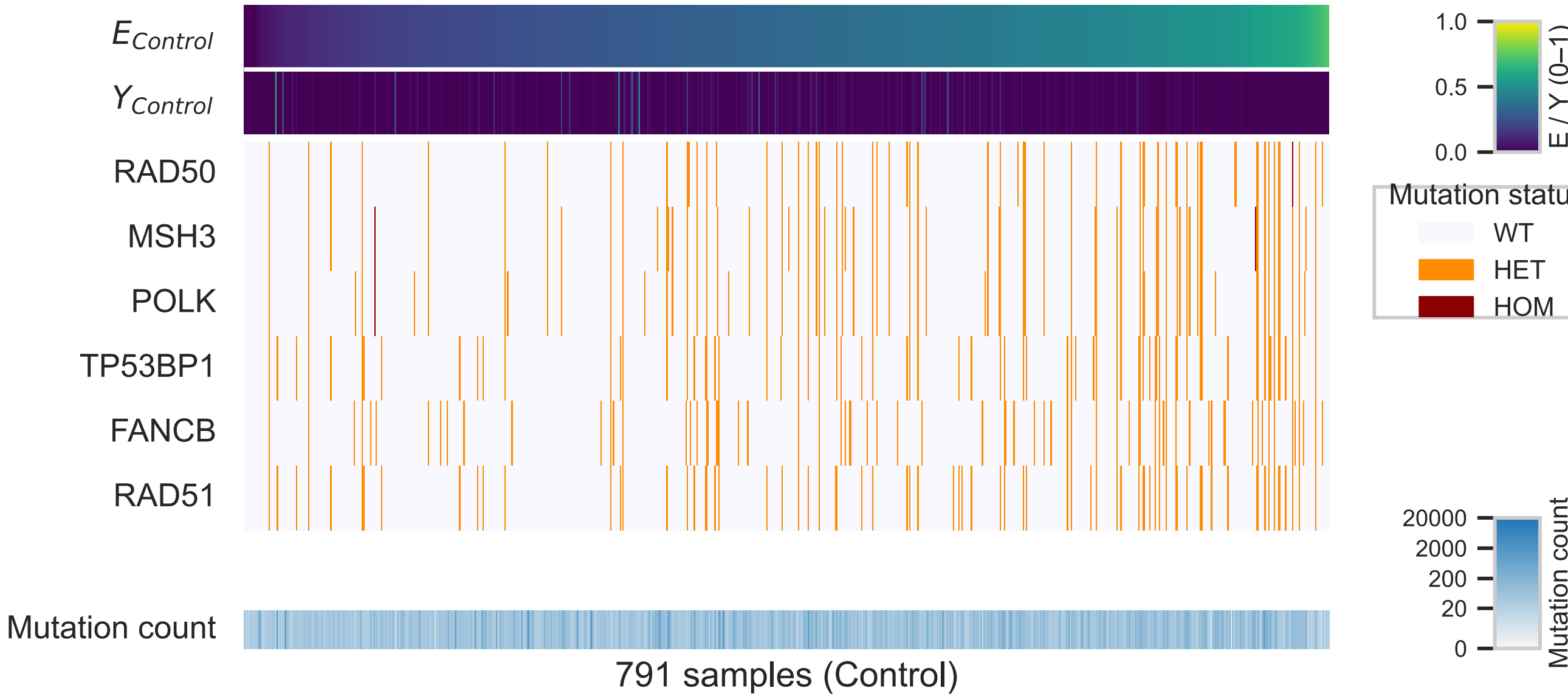

Fig. S30: **HR-associated tumour exposures of the non-integrated NMF control/background signature.** BRCA tumour samples are ordered by exposure to the non-integrated NMF control/background signature. Rows show signature exposure, mutation burden, and mutation status for HR-related genes significantly associated with this exposure. These genes overlap with the HR-associated genes identified for the SNMF HRd signature in the main analysis, suggesting that the unsupervised baseline captures related tumour variation but assigns it to a background/control-labelled signature.

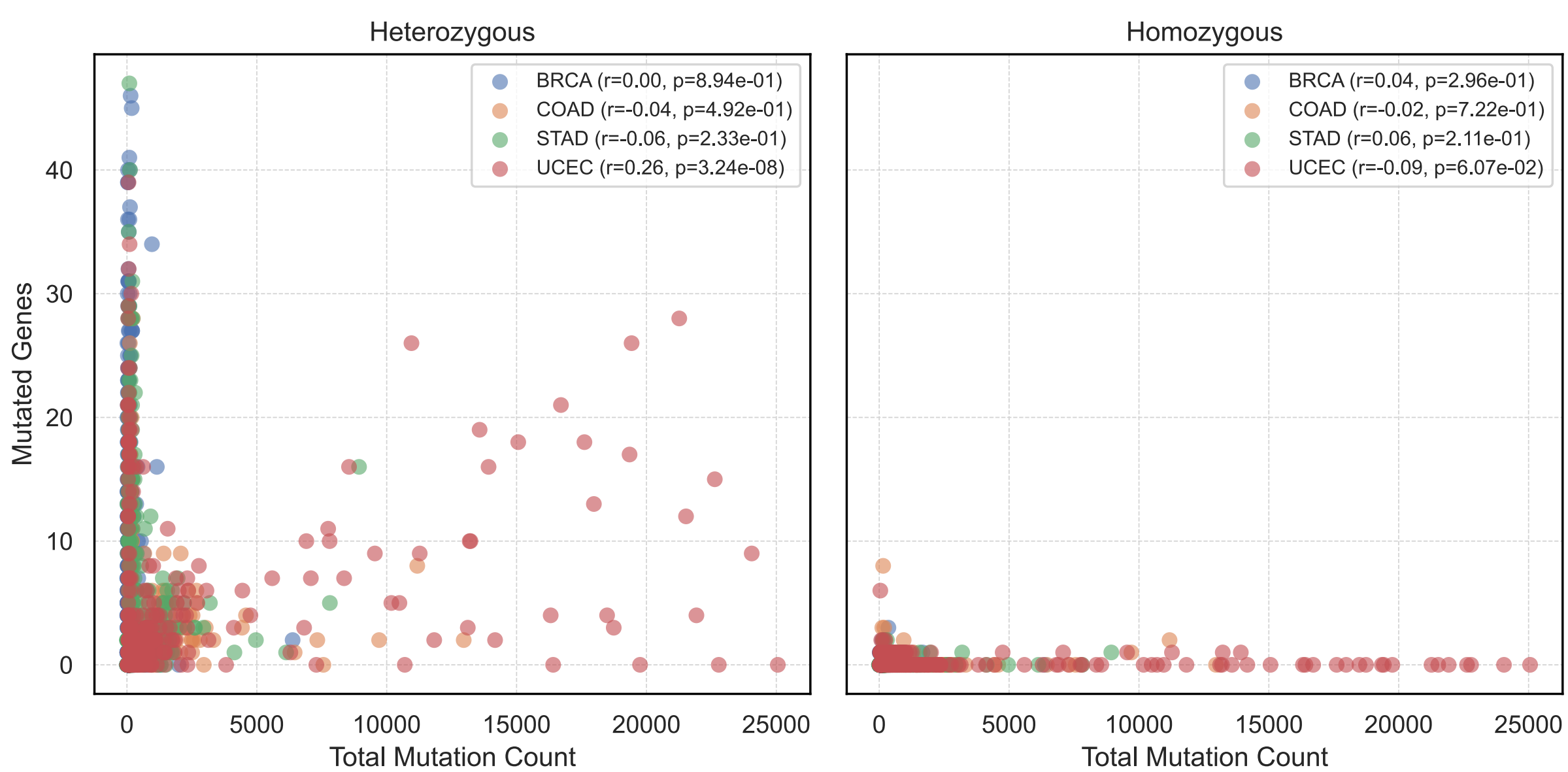

Fig. S31: **Relation between total mutation count and the number of DDR-related mutated genes across TCGA samples.** Considering either heterozygous (left) or homozygous (right) mutations to the DDR genes under analysis. Each point represents a tumour sample, colored by cancer type. Pearson correlation coefficients and p-values per cancer type are shown in the legend.

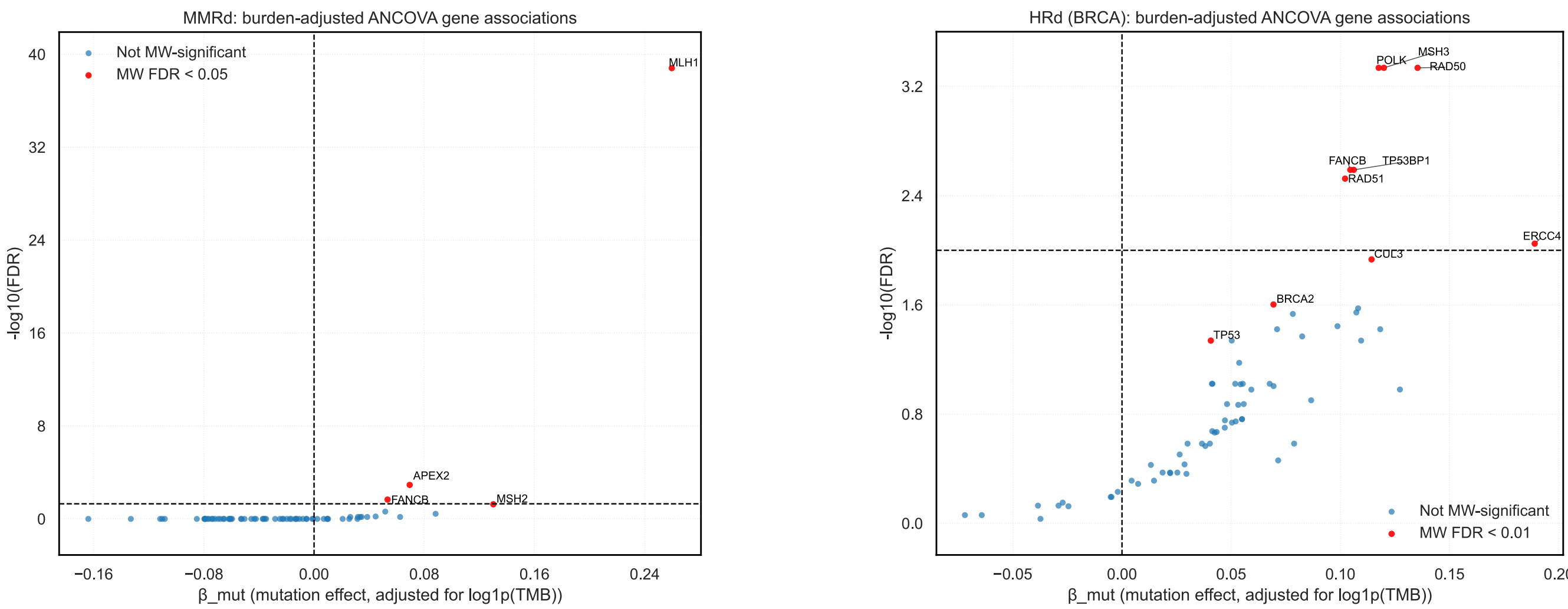

Fig. S32: **Burden-adjusted gene associations for DDR signatures (ANCOVA).** Volcano plots summarizing per-gene mutation effects on signature exposure after adjusting for tumour mutational burden (ANCOVA; covariate $\log(1 + \text{TMB})$) for **(left)** MMRd (SBS96B_MMR) ($p \leq 0.05$) and **(right)** HRd (SBS96A_HR; BRCA) ($p \leq 0.01$). Each point is a gene; the x-axis shows the estimated mutation effect (β_{mut}) and the y-axis shows $-\log_{10}(\text{FDR})$. Gene names are labeled if significant in either of the test (MW or ANCOVA).

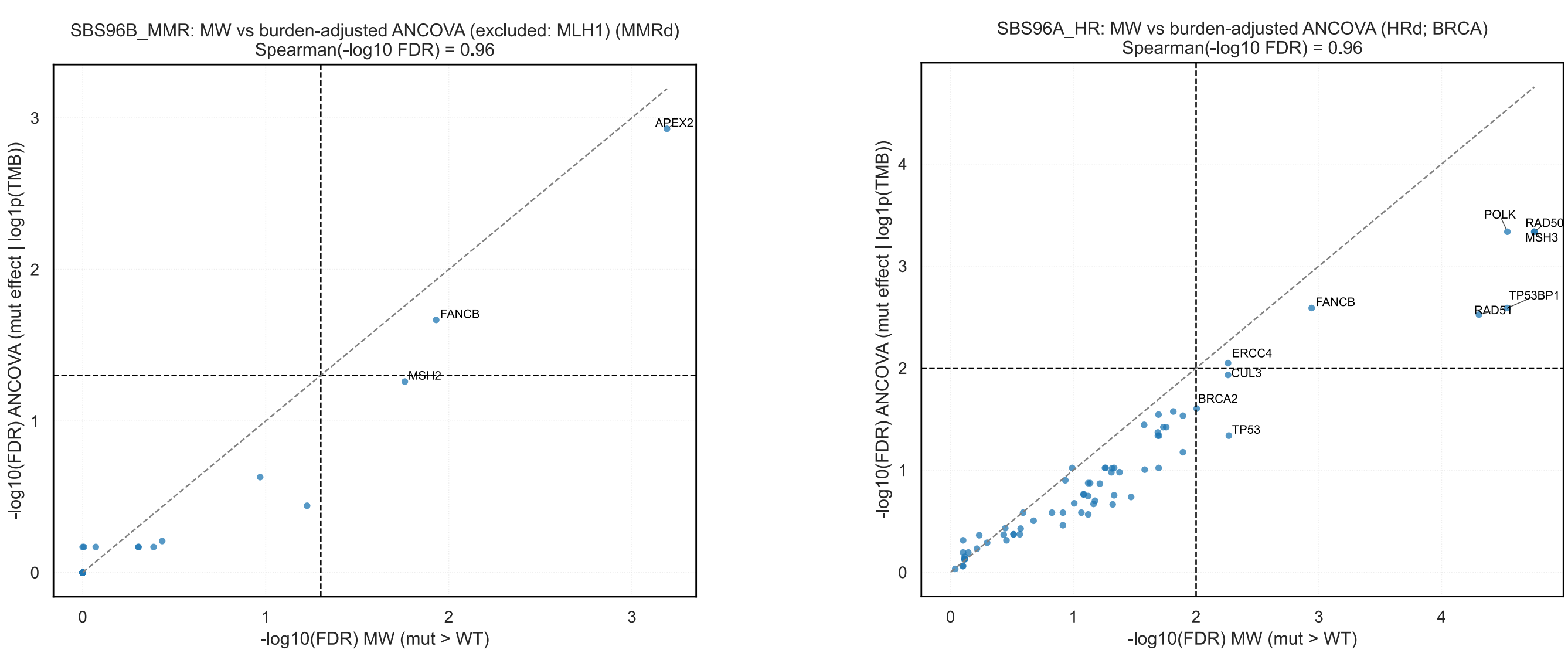

Fig. S33: **Consistency between MW and burden-adjusted ANCOVA gene associations.** Comparison of gene-level significance from Mann–Whitney U tests and ANCOVA for **(left)** MMRd (SBS96B_MMR; MLH1 excluded from the axis scaling) ($p \leq 0.05$) and **(right)** HRd (SBS96A_HR; BRCA) ($p \leq 0.01$). Each point is a gene; axes show $-\log_{10}(\text{FDR})$ from MW (x-axis) and ANCOVA (y-axis). Dashed lines indicate the FDR threshold used for significance, gene names are labeled if significant in either of the test.

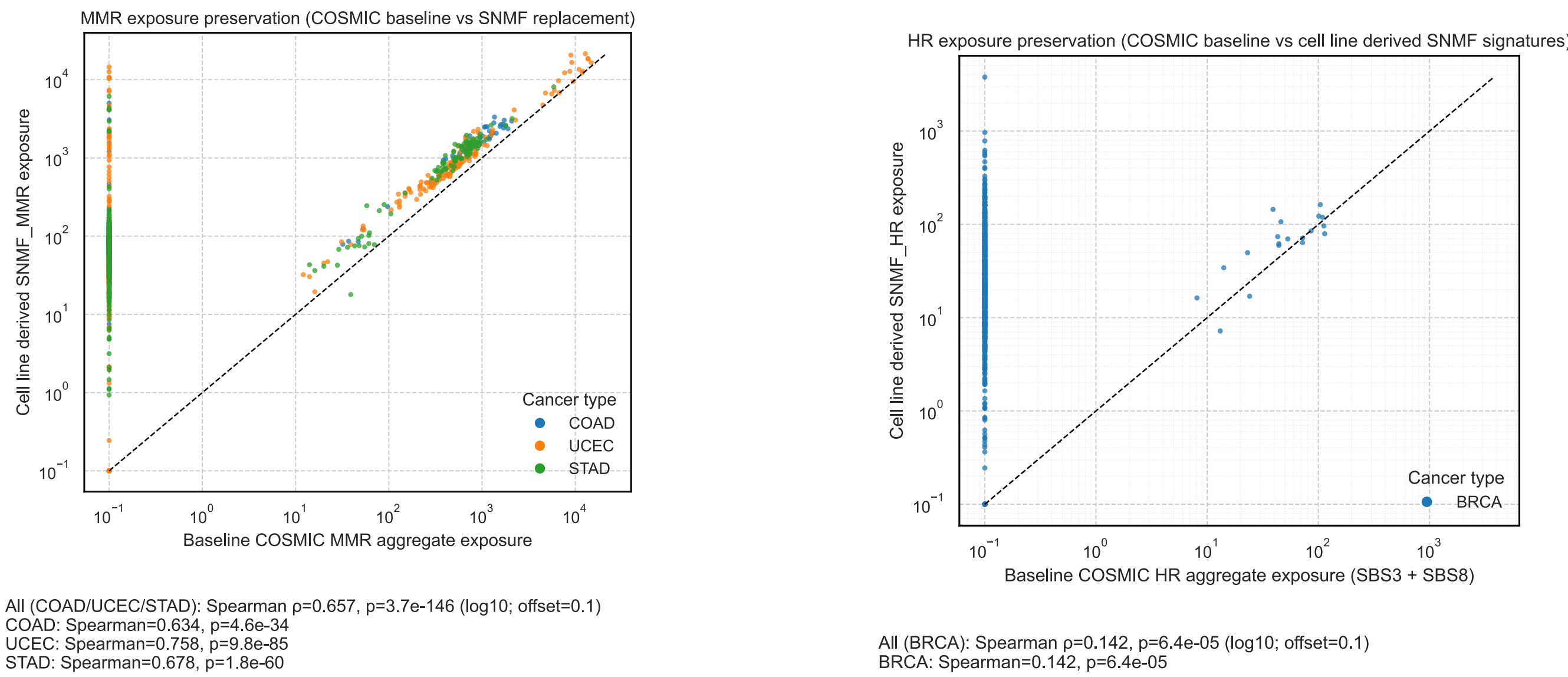

Fig. S34: **Preservation of DDR-associated exposures in tumours.** Comparison of baseline COSMIC aggregate exposures and cell line–derived SNMF exposures for **(left)** MMRd (All COSMIC SBS MMR aggregate vs. SNMF_MMR) and **(right)** HRd (SBS3+SBS8 aggregate vs. SNMF_HR, BRCA). Each point represents a tumour sample (log scale); dashed lines indicate $x = y$. Spearman correlations are shown in the panels.

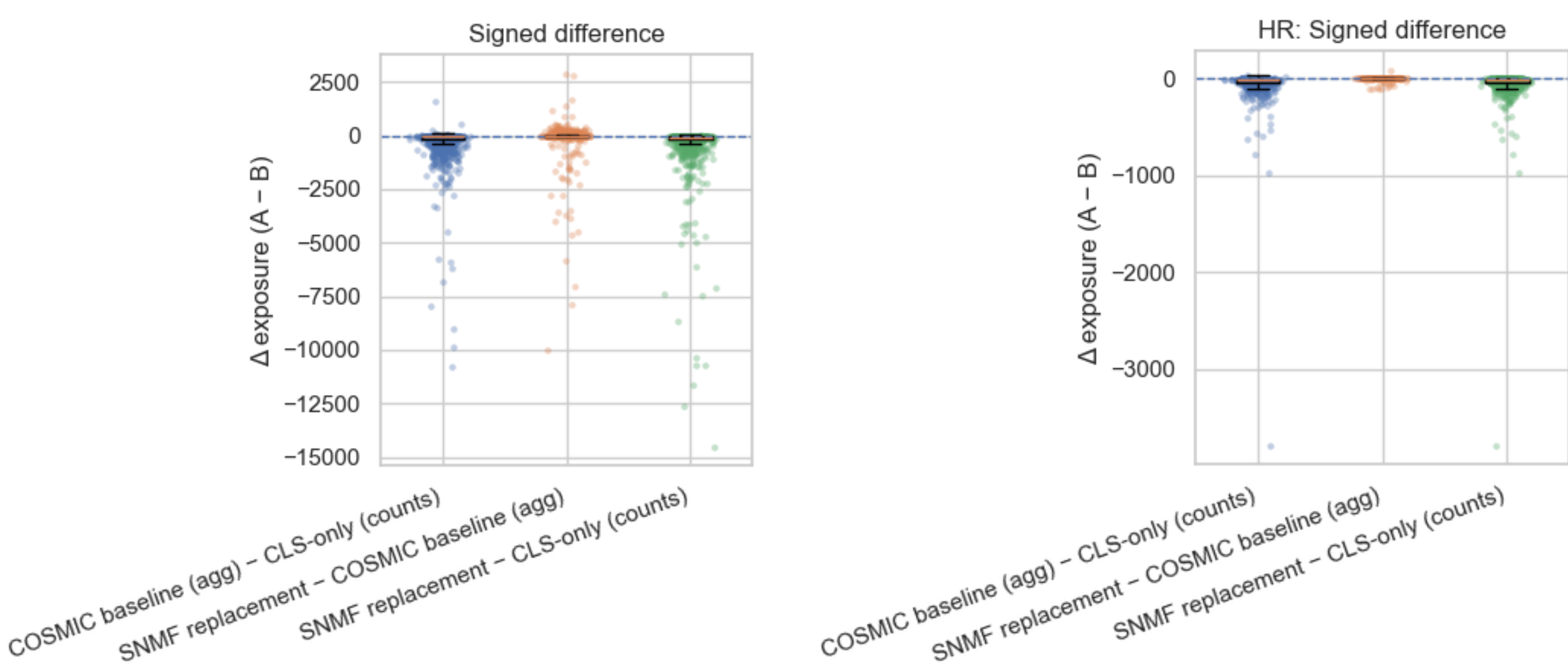

(A) MMRd-associated exposures.

(B) HRd-associated exposures.

Fig. S35: **Signed differences in tumour exposures across signature-fitting strategies.** Signed exposure differences are shown for **(A)** MMRd-associated and **(B)** HRd-associated tumour exposures across three pairwise comparisons of fitting strategies: restricted fitting of SNMF-learned cell-line signatures, fitting of the full COSMIC catalogue with aggregate DDR-associated COSMIC exposures, and custom fitting in which the corresponding COSMIC DDR-associated signatures were replaced by the SNMF-learned cell-line signatures. Positive values indicate higher exposure assigned by the first fitting strategy in the comparison, whereas negative values indicate higher exposure assigned by the second. These comparisons illustrate that absolute exposure estimates depend on the reference signature set used for fitting, supporting interpretation of transferred cell-line-signature exposures in relative terms rather than as prevalence estimates.
